# MXene-Assembled Liquid Metal Hybrid Microparticles for Multifunctional and Stretchable Printed Electronics

**DOI:** 10.1007/s40820-026-02154-3

**Published:** 2026-04-03

**Authors:** Rouhui Yu, Jiexin Qiu, Hui Zhu, Xiangheng Du, Jiale Sun, Zishuo Zhang, Long Chen, Zhongyao Fan, Huifang Chen, Meifang Zhu, Shaowu Pan

**Affiliations:** State Key Laboratory of Advanced Fiber Materials, College of Materials Science and Engineering, Donghua University, Shanghai, 201620 People’s Republic of China

**Keywords:** Liquid metals, MXene, Hybrid microparticles, Multifunctional electronics

## Abstract

Stretchable printed electronic devices are essential for the advancement of soft robotics, bioelectronics, and wearable systems. Liquid metals, owing to their high electrical conductivity and intrinsic deformability, have emerged as promising candidates for these applications. However, their limited functionality hinders their integration into multifunctional electronic devices. Here, we present versatile MXene-assembled liquid metal hybrid microparticles (MLHMs), which serve not only as conductive platforms for diverse electronic devices but also as electrochemical electrodes for stretchable energy storage devices. This multifunctionality stems from their unique structure, in which MXene nanosheets self-assemble around liquid metal microparticles via coordination interactions, forming an interconnected hybrid network within the printed pattern. This architecture enables the activation of electrical conductivity in hybrid microparticles at a minimal strain of 2.5%, achieving a high electrical conductivity of 3.7 × 10^5^ S m^−1^ and excellent stretchability of ~ 700%. The MLHMs demonstrate multifunctionality in stretchable antennas, micro-supercapacitors, electroluminescent devices, and flexible printed circuit boards, enabling wireless power transmission, energy storage, and stretchable and interactive display. These hybrids represent versatile material units for advancing stretchable and integrated electronic systems.

**Supplementary Information:**

The online version contains supplementary material available at 10.1007/s40820-026-02154-3.

## Highlights

- MXene-assembled liquid metal hybrid microparticles were developed by integrating MXene nanosheets with liquid metal particles through Ga–O–Ti coordination bonding.
- These hybrid microparticles can be precisely patterned onto diverse substrates via printing techniques, achieving high conductivity of 3.7 × 10^5^ S m^−1^.
- Their multifunctionality is demonstrated in applications including wireless power transmission, energy storage, and interactive display systems.

**Supplementary Information:**

The online version contains supplementary material available at 10.1007/s40820-026-02154-3.

## Graphical abstract

**Figure:**
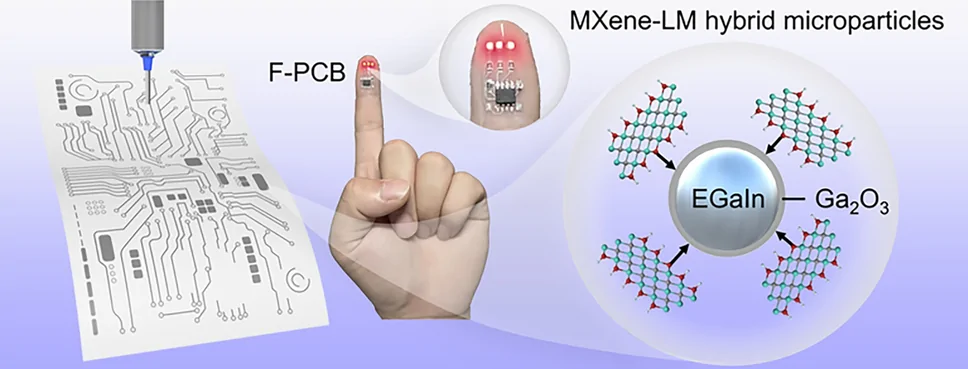

## Introduction

Stretchable electronics and systems have attracted significant attention for applications in soft robotics [1, 2], wearable devices [3–5], energy harvesting/storage [6, 7], and flexible display [8–10]. As essential building blocks of these systems, stretchable circuits are typically composed of conductive materials integrated with elastic substrates. To accommodate mechanical deformation, macroscopic stretchability is often achieved through structural engineering approaches. In particularly, rigid conductive materials, such as carbon nanomaterials and metal nanofilms, are often patterned into serpentine or wrinkled geometries on stretchable substrates to create stretchable devices [11–13]. However, the mechanical mismatch between these rigid materials and the soft substrates often leads to interfacial delamination during extended use, thereby compromising device durability.

Eutectic gallium-indium (EGaIn) liquid metal has emerged as a promising material for soft electronics due to its high electrical conductivity of 3.4 × 10^6^ S m^−1^, near-zero Young’s modulus, and excellent biocompatibility [14–17]. The shaping and patterning of liquid metal are crucial for tailoring its electrical properties and realizing multifunctional applications. Advanced printing technologies, including 3D printing, stencil printing, and inkjet printing, enable the scalable and efficient production of customized circuits for stretchable electronics [18–23]. However, EGaIn’s low wettability and high surface tension pose significant challenges for printing [24, 25]. Alternatively, liquid metal particles (LMPs), produced by ultrasonic treatment or mechanical stirring of bulk liquid metal, exhibit enhanced rheological properties and wettability, making them more suitable for printing technology [26, 27]. To further improve their processability and ink stability, LMPs are typically dispersed by hydrophilic polymers, such as sodium alginate, Carbopol, and poly(styrene sulfonate) [28–31]. The native oxide layer on the surface of LMPs interacts with polymer dispersant through hydrogen bonding, coordination bonding, and electrostatic interactions, resulting in stable and printable inks. However, although polymer dispersants can improve the stability and printability of LMP-based inks, they typically serve only as passive stabilizers and do not introduce additional electrical or electrochemical functionality. Likewise, while functional nanomaterials have been incorporated into LMP-based inks [32–34], most reports still emphasize electrical conductivity, and the broader functional potential of these additives remains largely unexplored.

As a prominent 2D conductive material, MXene exhibits attractive properties such as high electrical conductivity and a large specific surface area, making it a promising material for energy harvesting, energy storage, and sensing applications [35–38]. Moreover, their abundant terminal groups such as –O, –OH, and –F facilitate integration with other functional materials through hydrogen bonding and coordination bonding interactions [39, 40]. Such chemically active surfaces can improve dispersion and interfacial adhesion, thereby facilitating hybrid design and integration with other functional materials compared with many pristine carbon-based fillers [41, 42]. For example, MXene composites with functional additives such as poly(3,4-ethylenedioxythiophene):polystyrene sulfonate, reduced graphene oxide, and poly(vinyl alcohol) have been developed, exhibiting excellent properties such as good processability, high mechanical strength, and desirable electrical conductivity [43–45]. Despite these advantages, MXenes’ inherent non-stretchability restricts their applications in stretchable electronics. Specifically, the rigid 2D layered structure of MXenes leads to mechanical brittleness, resulting in crack formation or delamination under dynamic deformations. We envision that MXenes can be effectively integrated with LMPs as ideal composites for multifunctional applications. The resulting MXene-LM hybrid microparticles have at least two advantages: (1) The abundant surface groups of MXenes enable strong interfacial interactions with the oxide shells of LMPs, while the rigidity of MXenes facilitates efficient stress transfer, thereby activating the conductivity of LMPs even under low strain. (2) The two components exhibit highly complementary functionalities: the intrinsic stretchability of LM compensates for the mechanical brittleness of MXenes, while the high surface area and favorable electronic characteristics of MXenes contribute to improved electrochemical performance. While previous studies on LM–MXene composites have primarily focused on mechanical robustness or printability [46–48], the role of MXene-enabled interparticle bridging in enabling low-strain electrical activation of LMPs, as well as the electrochemical contribution of MXene within such composites, remains largely unexplored.

**Fig. 1:**
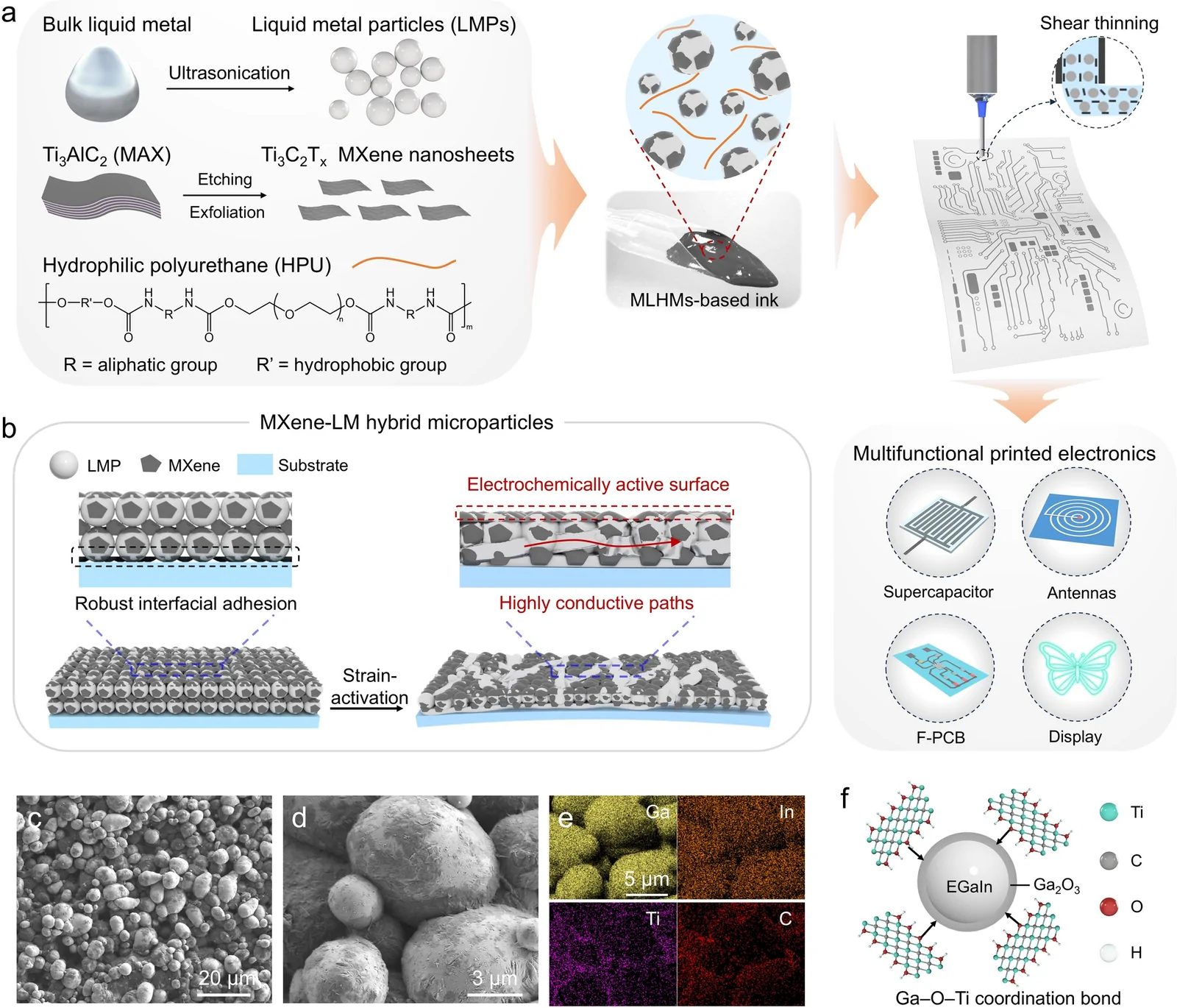
Design of MXene-assembled liquid metal hybrid microparticles (MLHMs) and their versatile applications in stretchable printed electronics. **a** Schematic illustration of the fabrication of MLHM-based ink and their versatile applications. **b** Diagram showing the characteristic and strain-activation process of MLHMs. **c, d** SEM image of MLHMs under different magnifications. **e** Element mapping images showing the distribution of Ga, In, Ti, and C elements within the MLHMs. **f** Schematic diagram of the coordination interaction between MXene and liquid metal

In this study, we developed MXene-assembled liquid metal hybrid microparticles (MLHMs) by combining LMPs and MXenes into an ethanol–water solution containing hydrophilic polyurethane (HPU). The MLHM-based ink shows viscoelasticity and shear-thinning behavior, enabling 3D and stencil printing of complex patterns on diverse substrates for multifunctional printed electronics (Fig. 1a). The assembled MXenes impart the printed MLHMs with enhanced electrochemical functionality and robust interfacial adhesion to the substrate. Within the hybrid microparticles, LMPs are interconnected by MXene bridges, enabling strain-activated electrical conductivity and superior performance (Fig. 1b). The printed pattern achieves a high conductivity of 3.7 × 10^5^ S m^−1^ under a minimal strain of 2.5% and exhibit stretchability of ~ 700%, which exhibits excellent comprehensive performance and multifunctional characteristics compared to those of previously reported printable and stretchable conductors (Table S1). The hybrid microparticles proved versatile for use in stretchable micro-supercapacitors (MSCs), electroluminescent devices, and antennas. As a proof of concept, we fabricated flexible printed circuit boards (F-PCBs) with interactive display capabilities, demonstrating the significant potential for integrated electronic systems.

## Experimental Section

### Materials

Eutectic gallium-indium alloy (EGaIn, 75% Ga and 25% In) was purchased from Shenyang Jiabei Trading Co., Ltd. Ti_3_AlC_2_ (400 mesh) was purchased from 11 Technology Co., Ltd. Hydrophilic polyurethane (HPU, HydroMed D3) was supplied by AdvanSource Biomaterials. Thermoplastic polyurethane (TPU, Elastollan 1185 A) was provided by BASF Co. Ltd. Polydimethylsiloxane (PDMS, Sylgard184) was purchased from Dow Corning. Lithium fluoride (LiF), Poly(vinylidene fluoride-co-hexafluoropropylene) (PVDF-HFP) and 1-Ethyl-3-methylimidazolium bis(trifluoromethylsulfonyl)imide ([EMIM][TFSI]) was purchased from Shanghai Titan Technology Co., Ltd. Hydrochloric acid (HCl) and acetone was purchased from Sinopharm Chemical Reagent Co., Ltd. Silver nanowires (AgNWs, diameter: 70 nm, length: 45 μm) was purchased from Shanghai Aladdin Biochemical Technology Co., Ltd. ZnS:Cu phosphor powder was purchased from Shanghai Keyan Optoelectronics Technology Co., Ltd.

### Fabrication of Ti_3_C_2_T_***x***_ (MXene) Nanosheets

Ti_3_C_2_T_*x*_ (MXene) nanosheets were prepared by selective etching of Al layer from Ti_3_AlC_2_ MAX phase powder with HCl and LiF. Typically, 2 g LiF powder was added into a Teflon beaker containing 40 mL 9 M HCl and stirred for 10 min. Subsequently, 2 g Ti_3_AlC_2_ powder was slowly added and stirred continuously for 24 h at 35 °C. The final reaction mixture was washed with deionized water and centrifuged at 3500 rpm for 10 min for several times until the pH of the supernatant was above 6. The sediment was re-dispersed in 40 mL deionized water for ultrasonic 1 h, and then the few-layer of MXene dispersion was obtained by centrifuging at 1500 rpm for 3 min to separate the dark green supernatant. The delaminated few-layer MXene nanosheets were obtained by lyophilization.

### Fabrication of MLHMs-Based Ink

Typically, 2 g of bulk liquid metal (LM) was added to 1.5 g of a 1% (w/v) hydrophilic polyurethane (HPU) solution (ethanol:deionized water = 95:5, v/v). The mixture was tip-sonicated (JY92-IIN, Scientz, 6 mm microtip) at 300 W for 3 min. Subsequently, the mixture was centrifuged to remove the HPU solution, isolating the liquid metal particles. These particles were then added to 1.2 g of MXene/HPU solution and stirred continuously for 10 min to produce printable conductive ink. The MXene/HPU solution was prepared by mixing 0.5 g of MXene aqueous dispersion (40 mg mL^−1^) with 0.7 g of 1% (w/v) HPU solution. During ink formulation, several precautions were taken to minimize MXene oxidation. Specifically, MXene was stored under an inert atmosphere and kept in sealed containers prior to use, and the ink was prepared fresh and used immediately to further reduce exposure to oxygen. The key ink fabrication steps, including sonication and lyophilization, are compatible with scalable industrial processes. However, maintaining process consistency remains an important consideration for large-scale production.

### Printing of MLHMs-Based Ink

For direct ink writing (DIW) 3D printing process, a commercial printer (BioScaffolder Prime, GESIM) was employed. The prepared ink was loaded into a syringe fitted with a 240 μm nozzle. During printing, the nozzle maintained a 0.5 mm vertical distance from the substrate, with a movement speed of 20 mm s^−1^. Printing paths were designed using computer-aided design software (3ds Max 2021). For stencil printing, a 60 μm low-adhesion PET tape was used as a mask, patterned with a CO_2_ laser cutter (Venus V-12, GCC LaserPro) at 12 W. The mask was applied to the substrate, and the MLHM-based ink was spread over it using a rubber scraper. After drying at room temperature, the mask was removed from the substrate to obtain MLHMs pattern.

### Fabrication of Multifunctional and Stretchable Printed Electronics

#### Fabrication of Antenna

The antenna was fabricated on a TPU substrate using DIW 3D printing, forming a coil with a 4 cm diameter, 1 mm line width, and 1 mm gap. A straight line was printed on a separate TPU film. Vertical interconnect accesses (VIAs) were engineered between the two layers of the 3D liquid metal (LM) circuits and filled with LM. A micro-LED was attached to the two leads and secured with adhesive.

#### Fabrication of the Stretchable Electroluminescent Device

The LM pattern was fabricated on PDMS substrates via DIW 3D printing MLHMs as bottom electrode of electroluminescent device. The PDMS substrate was modified using an amino-functional silane coupling agent to introduce –NH_2_ groups onto the surface. This treatment converts the inherently hydrophobic PDMS surface into a more hydrophilic and chemically active interface, which significantly enhances interfacial adhesion with the MLHMs. Specifically, PDMS films were activated by oxygen plasma treatment (100 W, 3 mbar; SY-DT01) for 3 min, then immersed in 100 mL of silane solution (10 µL acetic acid, 1% (w/v) 3-aminopropyltrimethoxysilane in deionized water) for 1 h at room temperature. The treated films were rinsed with deionized water and dried under a nitrogen flow. A luminescent layer with a thickness of ~ 70 μm was formed by spin-coating the ZnS:Cu/PDMS mixture onto the films, followed by thermal curing. Prior to top electrode deposition, the composite film was heated to 80 °C to facilitate rapid evaporation of the ethanol solvent. A 0.5 mg mL^−1^ AgNWs/ethanol solution was then sprayed onto the surface to form the top electrode. Finally, a PDMS encapsulation layer was applied via spin-coating to complete the stretchable electroluminescent devices.

#### Fabrication of Energy Storage Devices

The MLHMs interdigitated electrode pattern was fabricated via stencil printing, with both line width and interelectrode gap of 500 μm. Flexible MSC devices were then prepared by casting an ion gel solution onto the MLHMs electrode region (effective area: 1 cm^2^), followed by drying in an oven at 50 °C. The ion gel electrolyte was prepared by mixing [EMIM][TFSI] with PVDF-HFP. Briefly, 0.3 g of PVDF-HFP was dissolved in 5 mL of acetone, after which 1.8 g of [EMIM][TFSI] was added. The mixture was stirred at 50 °C for 8 h to obtain a homogeneous ion gel.

#### Fabrication of Stretchable LED Array

Customized MLHMs interconnects forming the letters “LMP” were fabricated on TPU substrates via stencil printing. A total of 26 LEDs were integrated onto the pattern to form the LED array. To enhance adhesion, the LEDs were affixed using glue. The array was powered by an applied voltage of 3 V.

#### Fabrication of Flexible Printed Circuit Board (F-PCB)

MLHM-based circuits were fabricated on TPU substrates by stencil printing. A commercial integrated chip (NE555) and electronic components (including three 1 kΩ fixed resistors, one 22 kΩ fixed resistor, a photoresistor and three LEDs) were integrated with the printed patterns to realize a light-controlled LED switching circuit.

### Characterization

The surface morphology of printed line was observed using cold field emission scanning electron microscope (SEM, SU-8010, Hitachi). Fourier transform infrared spectroscopy (FTIR) was recorded on a Fourier transform infrared spectrometer (Nicolet iS50, PerkinElmer). The electrical properties were characterized by a source-meter (2461, Keithley). Deformation control of samples was conducted by universal tensile testing machine (UTM2103, Shenzhen Suns technology). X-ray diffraction (XRD) was performed to evaluate the structure of materials using an XRD diffractometer (D8 Advance, Bruker). X-ray photoelectron spectroscopy (XPS) was carried out using an X-Ray photoelectron spectrometer (K-Alpha, Thermo Scientific). Electrochemical measurements of energy story devices were carried out by an electrochemical workstation (CHI 660E, Shanghai Chenhua co.). All human skin attachment experiments were approved by the ethics committee of Donghua University (No. DHUEC-NSFC-2023–49). Consent from all participants was obtained for the use of electronic device on human skin before the study commenced. The electrical conductivity was calculated based on the measured resistance, sample length, and cross-sectional area according to *σ* = *L*/(*RA*), where *L* is the length of the tested sample (1 cm), *R* is the sample resistance, and *A* is the cross-sectional area calculated as the product of the line width and thickness. The line width and thickness of each sample were determined using optical microscopy. For thickness measurement, three representative regions were measured, and the average value was used to calculate the cross-sectional area. Conductivity values were obtained from five independently fabricated samples (*n* = 5) and are presented as mean ± standard deviation.

## Results and Discussion

### Design and Characterization of MLHMs-Based Ink

The MLHM-based ink was formulated by incorporating LMPs, MXene nanosheets, and HPU into an ethanol–water mixed solvent (Fig. S1). LMPs with an average diameter of ~ 3.4 μm were obtained by tip sonication of bulk EGaIn in an HPU solution (Fig. S2). Notably, the presence of HPU significantly improved the size uniformity of the LMPs after sonication. Compared to LMPs formed in ethanol without HPU, those stabilized by HPU exhibited a narrower particle size distribution, confirming the stabilizing effect of the polymer (Fig. S2). The FTIR spectra show that the carbonyl (–CO–) stretching band shifts from 1696 cm^−1^ in the HPU polymer to 1705 cm^−1^ in the HPU/LMP (Fig. S3). This blue shift indicates the presence of electrostatic interactions between the LMPs and HPU. In addition, elemental mapping of the HPU/LMP composite shows that carbon originating from HPU is distributed around the LMPs, providing further evidence that HPU is adsorbed onto the particle surfaces through electrostatic interactions (Fig. S4). MXene nanosheets were prepared by selectively etching Ti_3_AlC_2_ using HCl/LiF solution, followed by exfoliation (Fig. S5). The successful synthesis of 2D MXene nanosheets was evidenced by the shift of X-ray diffraction peak (002) from 9.6° to 5.7° and the disappearance of the Al (104) diffraction peak of Ti_3_AlC_2_ (Fig. S6) [49, 50]. The obtained MXene nanosheets had an average lateral size of ~ 517 nm and a thickness of ~ 3.5 nm (Fig. S7). X-ray photoelectron spectroscopy (XPS) spectra confirm the presence of terminal groups such as C–Ti–F_*x*_, C–Ti–(OH)_*x*_, and C–Ti–O_*x*_ (Fig. S8). Owing to the abundant hydrophilic functional groups, MXenes are well dispersed in the aqueous solvent without sedimentation. LMPs are added to MXenes at a 100:1 mass ratio, where coordination between Ga^3+^ ions and oxygen-containing groups on MXenes leads to the formation of MXene-LM hybrid microparticles (MLHMs) [46, 47]. However, these MLHMs tend to sediment under gravity. To enhance ink stability, HPU with a concentration of 7.5 mg mL^−1^ is introduced as a polymeric stabilizer. The presence of HPU significantly enhances the dispersion stability, with no noticeable sedimentation observed after 12 h (Fig. S9). This stabilization arises from the stabilizing effect of HPU through increasing the ink viscosity, as well as from the hydrogen-bond interactions between HPU and the functional groups on MLHMs, including HPU–NH···O–MXene, HPU–NH···F–MXene, and HPU–CO···HO–MXene. The hydrogen bonding interaction between MXene nanosheets and HPU is confirmed by infrared spectroscopy. The characteristic hydroxyl stretching vibration of MXene at 3456 cm^−1^ shifts to a lower wavenumber of 3338 cm^−1^ in the presence of HPU, indicating strong hydrogen bonding interactions between HPU and MXene nanosheets (Fig. S10).

**Fig. 2:**
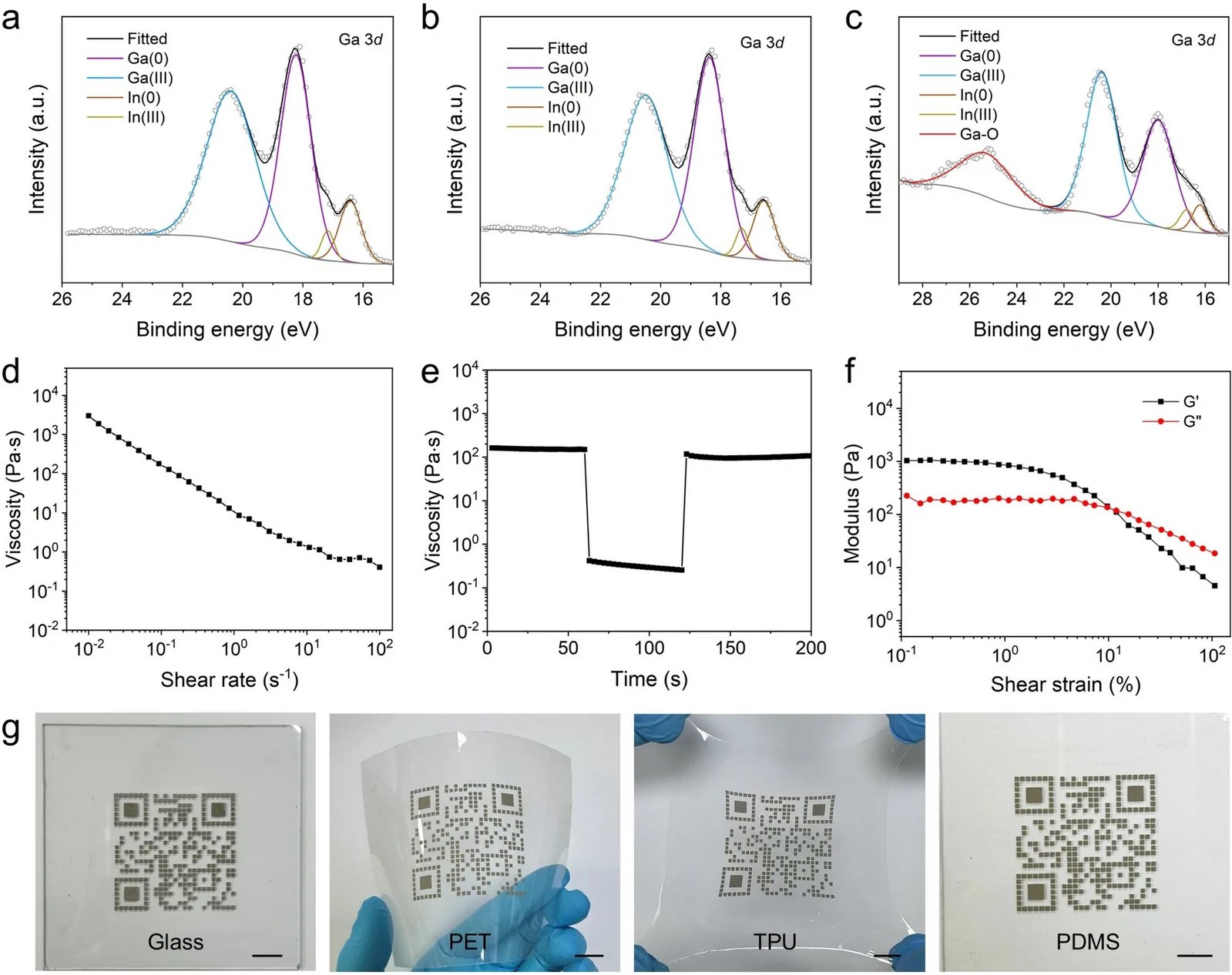
Characterization and patterning capability of MLHMs. High-resolution Ga 3*d* XPS spectra of **a** pure LMPs, **b** LMPs/HPU composites, and **c** LMPs/HPU/MXene composites. **d** Viscosity of MLHM-based ink as a function of shear rate. **e** Viscosity as a function of shear rate with three stages. **f** Variation of *G'* and *G''* as a function of oscillating strain. **g** Stencil-printed QR code patterns on various substrates, including glass, PET, TPU, and NH_2_-functionalized PDMS; the scale bar is 1 cm

The microscopic morphology of MLHMs after solvent evaporation was characterized using scanning electron microscopy (SEM). The SEM images show that MXene nanosheets are well assembled with LMPs to form hybrid microparticles (Fig. 1c). In the magnified view, the MXene nanosheets bridge adjacent LMPs, forming the interconnected network (Fig. 1d). Elemental mapping further confirms that MXene nanosheets partially wrapped around the LMPs (Fig. 1e). This hybrid structure is attributed to the formation of Ga–O–Ti coordination bonds between the negatively charged surface functional groups of the MXene and Ga^3+^ from the Ga_2_O_3_ passivation layer on the EGaIn particles (Fig. 1f). After washing away the surface HPU, the EDS mapping still shows MXene attached to the LMP surface, indicating that MXene remains firmly bonded to LMPs (Fig. S11). Figure 2a**–**c shows the Ga 3*d* X-ray photoelectron spectroscopy (XPS) spectra of pure LMPs (Fig. 2a), LMPs/HPU composites (Fig. 2b), and LMPs/MXene/HPU composites (Fig. 2c). The presence of HPU does not significantly alter the peak position or width compared to pure LMPs, indicating a lack of chemical interaction between the Ga in the LMPs and HPU. In contrast, the Ga 3*d* spectrum of the LMP/MXene/HPU composites exhibits an additional Ga–O peak at 25.3 eV, which is absent in pristine LMPs and LMPs/HPU composites. Although physical adsorption or residual MXene may contribute to interfacial interactions, simple adsorption alone cannot explain the selective emergence of this peak in the MXene-containing composite. This feature suggests the formation of a Ga–O–Ti bridging environment, suggesting strong interfacial interactions between LMPs and MXene nanosheets [44, 51, 52].

To evaluate the printability of MLHM-based ink, its rheological properties were characterized using a rotational rheometer. The viscosity-shear rate curve demonstrates typical shear-thinning behavior, with an initial viscosity of ~ 3.0 × 10^3^ Pa s (Fig. 2d). As the MXene content increases, the ink exhibits higher viscosity, likely due to the formation of more cross-linked networks by MXene bridges (Fig. S12). A peak-hold step test was conducted to simulate the shear response and recovery behavior during extrusion-based 3D printing or stencil printing. In this test, the ink was subjected to three distinct shear rate intervals. Initially, at a low shear rate of 0.1 s^−1^ for 50 s, the ink maintained a high viscosity of 150.1 Pa s. The shear rate was then abruptly increased to 100 s^−1^ for 50 s to mimic extrusion or scrape-coating, causing the viscosity to drop sharply to 0.4 Pa s. When the shear rate was returned to 0.1 s^−1^, the viscosity rapidly recovered to 118.0 Pa s, achieving a recovery rate of 79% (Fig. 2e). This fast recovery time and high recovery rate are primarily attributed to the rapid deconstruction and reconstruction of dynamic cross-linked networks among hybrid microparticles. The viscoelastic behavior of the ink was further investigated by measuring the storage modulus (*G'*) and loss modulus (*G''*) as functions of the oscillatory strain (Fig. 2f). At low-strain levels, where *G'* > *G''*, the ink exhibits solid-like behavior. As the strain increases, the cross-linked network is disrupted, leading to *G'* < *G''*, which indicates a transition to liquid-like behavior that facilitates the printing process. The MLHM-based ink exhibits good wettability on a variety of substrates, while the resulting MLHMs demonstrate strong interfacial adhesion with these substrates, as qualitatively evaluated by tape-peeling tests (Figs. S13 and S14). As a result, the MLHM-based ink can be readily patterned on diverse substrates. For example, this ink was successfully stencil-printed in a quick response (QR) code pattern onto various substrates, including glass, stretchable TPU films, flexible PET, and amino-functionalized PDMS films (Fig. 2g). For DIW printing, increasing the printing speed resulted in a decrease in the linewidth, with a minimum achievable value of ~ 150 μm (Fig. S15). Furthermore, we demonstrated reproducible printing across multiple regions (Fig. S16a and Video S1). The printed patterns remain intact under bending deformation without observable delamination, indicating robust adhesion to the substrate (Fig. S16b). The optical images further reveal well-defined and continuous edges for patterns printed at different extrusion speeds, confirming good line-edge fidelity (Fig. S16c, d).

### Activation of the Conductivity of MLHMs

**Fig. 3:**
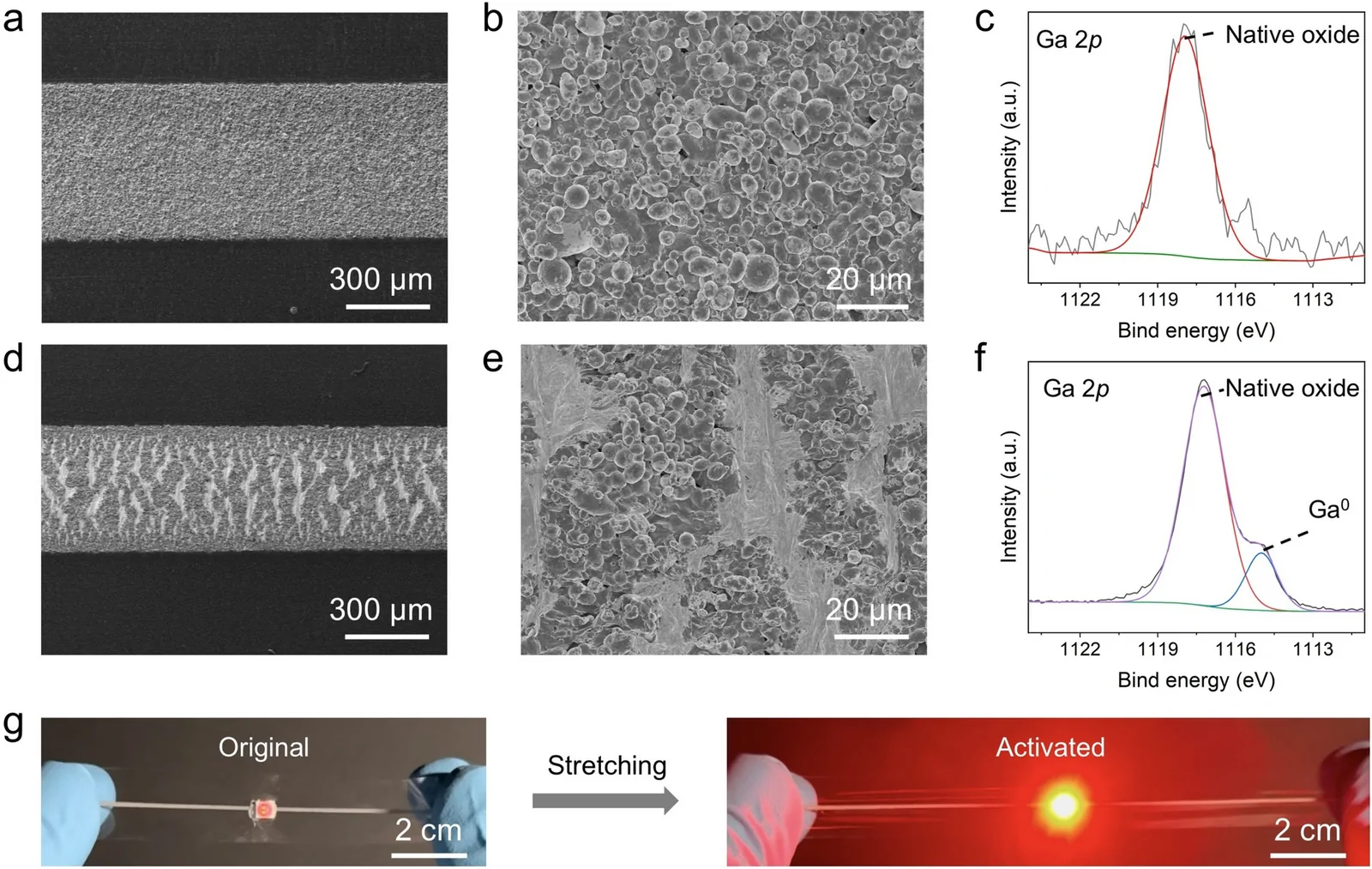
Strain-activation mechanism of MLHMs. **a** SEM image of the printed MLHMs line in the initial state. **b** Magnified view of (**a**). **c** High-resolution Ga 2*p* XPS spectrum of MLHMs before strain activation. **d** SEM image of the MLHMs under 50% uniaxial strain. **e** Magnified view of (**d**). **f** High-resolution Ga 2*p* XPS spectrum after strain activation. **g** Photographs of an LED connected to MLHMs before and after stretching. The LED was powered by 3 V direct current source

The formation of a cross-linked network among LMPs is critical for achieving low-strain activation of conductive pathways [53, 54]. In the printed MLHMs, LMPs are densely packed, with their surfaces partially wrapped and interconnected by MXene nanosheets, forming a continuous cross-linked network (Fig. 3a, b). In contrast, pure LMPs pack loosely with noticeable interparticle voids during solvent evaporation owing to the absence of interfacial bridging (Fig. S17). Before strain activation, the Ga 2*p* XPS spectrum of MLHMs shows only a characteristic peak corresponding to gallium oxide (Fig. 3c). Upon applying 50% tensile strain, continuous LM stripes appear along the direction of deformation, indicating strain-induced rupture of the oxide shells (Fig. 3d, e). This behavior is attributed to the strong interfacial interactions between the LMPs and MXene nanosheets, which facilitate efficient stress transfer from the rigid MXene to the Ga_2_O_3_ shells. As a result, the encapsulated liquid metal cores are released, forming conductive pathways. The appearance of a distinct elemental gallium peak in the Ga 2*p* XPS spectrum under strain further confirms the exposure of metallic Ga and the formation of conductive pathways (Fig. 3f).

The strain-activation process is also clearly observable from the back side of the composite. In the unstrained state, the LMPs appear as discrete particles. After applying 5% strain for 10 cycles, partial coalescence is observed. With further deformation up to 100% strain, a more pronounced merging of the LMPs occurs, indicating strain-induced a continuous LM phase (Fig. S18). The strain-activated conductivity of MLHMs can also be qualitatively demonstrated using a printed MLHMs line integrated into a LED circuit (Fig. 3g and Video S2). When a 3 V DC voltage is applied, the LED initially emits low brightness due to the presence of insulating Ga_2_O_3_ shells, which prevent the formation of continuous metallic pathways. At this stage, electrical conduction is primarily provided by the MXene nanosheets wrapping the LMPs. Upon applying slight tensile strain, the LED brightness increases significantly, indicating the rupture of the oxide shells and the formation of highly conductive LM pathways. In addition to strain activation, freezing can also effectively activate the conductivity of MLHMs. A 3 cm-long printed line on glass initially exhibits a high resistance of 27 kΩ. After freezing in liquid nitrogen, the resistance significantly decreases to 18 Ω (Fig. S19). This phenomenon is attributed to the expansion of liquid metal at low temperatures, which causes rupture of the oxide shells and promotes coalescence of LMPs [55, 56]. Consequently, this freeze-activation strategy enables the formation of highly conductive pathways even on rigid, non-stretchable substrates such as glass, silicon wafers, and metal surfaces.

The interface between the active materials and the stretchable substrate is crucial for achieving stable and reliable device performance. We investigated the interfacial property of three types of coatings on the TPU substrates, including LMPs, LMPs/HPU, and LMPs/MXene/HPU (MLHMs/HPU). After solvent evaporation, cracks appeared in both the LMPs and LMPs/HPU coatings, likely caused by weak interparticle interactions. In contrast, the MLHMs/HPU coating exhibited a uniform and crack-free morphology (Fig. S20). Tape-peeling tests further demonstrated that the MLHMs/HPU coating exhibited significantly stronger adhesion to the substrate compared to coatings without MXene (Fig. S20). The LMPs coating was readily peeled off due to poor interfacial adhesion. Even with a small amount of HPU, most of the coating detached during the peeling process. For the MLHM-based coating, only a thin surface layer consisting of MXene and a small amount of LMPs was partially removed, while the majority of the coating remained firmly attached to the substrate. This enhanced adhesion is attributed to the hydrophilic functional groups on MXene nanosheets, which improve interfacial compatibility. Additionally, MXene nanosheets fill the gaps between LMPs, thereby increasing the effective contact area between the coating and the substrate. The strong adhesion was further confirmed by optical images of the MLHMs/HPU coating under tensile deformation. The coating remained firmly attached to the TPU film without visible delamination. In contrast, the pure LMP coating, lacking interparticle bridging and strong interfacial bonding, showed obvious cracking and delamination from the substrate under strain (Fig. S21).

**Fig. 4:**
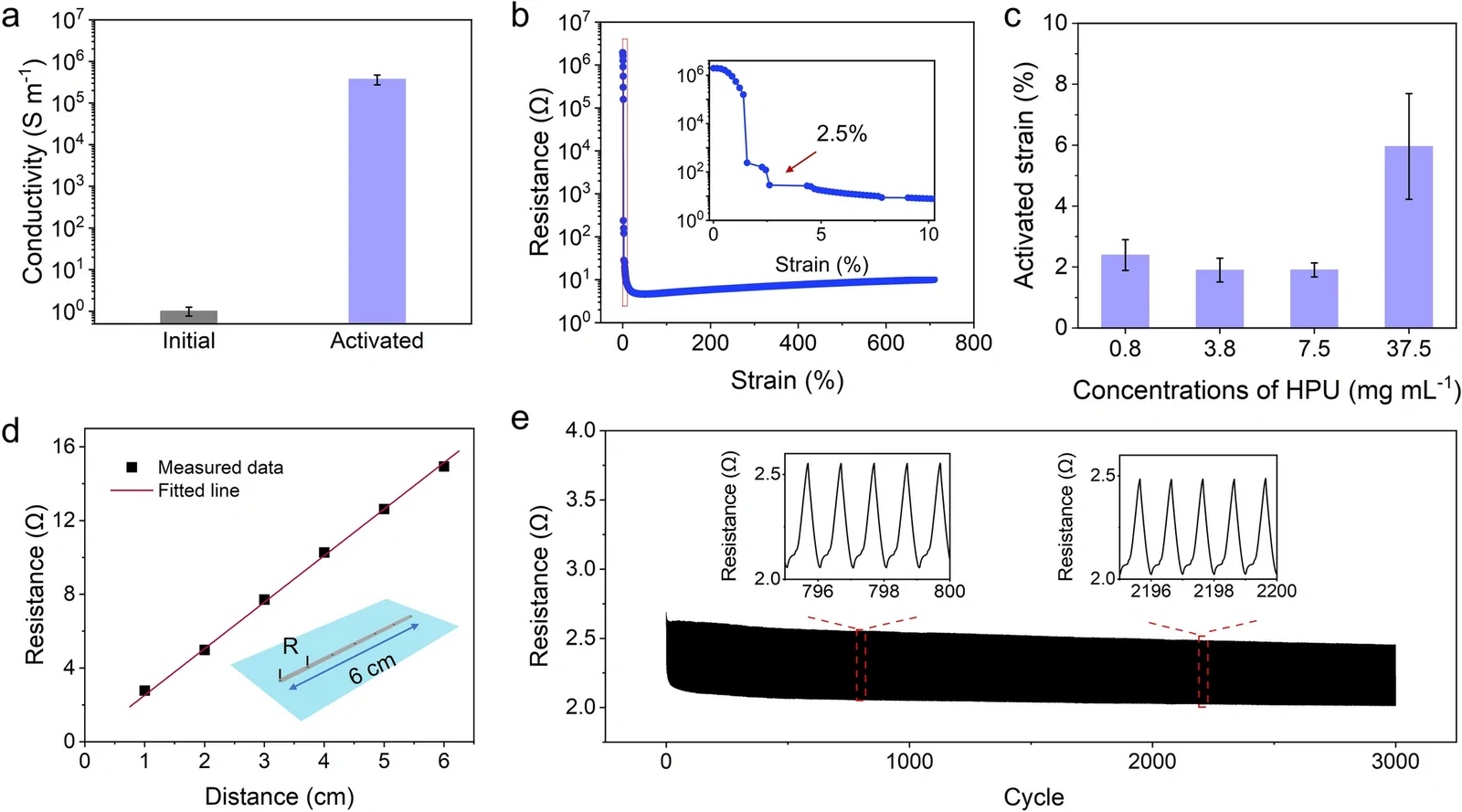
Electrical properties of MLHMs. **a** Comparison of the electrical conductivity of M_1_L_100_HMs before and after mechanical activation. The activation process was performed by applying 100% strain for 10 cycles. Data are presented as mean values ± s.d. (*n* = 5 independent samples). **b** Resistance of MLHMs under uniaxial tensile strain. **c** Activated strain of MLHMs with different concentrations of HPU. **d** Uniformity of electrical resistance over a total length of 6 cm. **e** Resistance of MLHMs under 100% cyclic strain over 3000 stretching-releasing cycles

The conductivity of MLHMs was quantitatively measured before and after activation (Fig. 4a). Prior to activation, the conductivity of M_1_L_100_HMs (MXene: LMP mass ratio of 1:100) was 1.0 S m^−1^. After strain activation, the conductivity dramatically increased to 3.7 × 10^5^ S m^−1^, representing an impressive five order of magnitude. With an increase of MXene content, the initial conductivity of M_2_L_100_HMs increased to 9.0 S m^−1^, while the post-activation conductivity remained nearly unchanged at 3.7 × 10^5^ S m^−1^ (Fig. S22). Given the negligible difference in final performance, M_1_L_100_HMs was selected for subsequent studies. The changes in the electrical resistance of MLHMs during the stretching process are shown in Fig. 4b. We define the activation strain as the strain at which the conductor resistance falls below 100 Ω. Remarkably, MLHMs achieve activation at a low strain of ~ 2.5%, with their resistance decreasing by four orders of magnitude. While previous studies have investigated strain-induced activation in polymer-modified LMPs, MLHMs achieve activation at a much lower strain (Table S2). This exceptional performance is attributed to the rigid MXene nanosheets, which efficiently transmit stress among MLHMs under tensile strain, compared to previously reported LMPs functionalized with polymers. Upon further stretching to 700% strain, the resistance remained below 10 Ω, demonstrating excellent stretchability. Beyond this strain, electrical failure was caused by mechanical rupture of the TPU substrate rather than degradation of the conductive network. In addition, the activation process of MLHMs was quantitatively characterized based on their electromechanical performance (Fig. S23). A larger applied tensile strain led to a more complete activation of electrical conductivity. Under 100% strain, the conductivity was nearly fully activated after 10 loading–unloading cycles. Further increasing the strain to 200% did not result in a significant additional decrease in resistance, indicating that the activation process had essentially reached saturation.

We further studied the effect of varying HPU content on the activation strain of the composites (Fig. 4c). When the HPU concentration was below 7.5 mg mL^−1^, the composites exhibited a low activation strain of less than 3%, indicating that the polymer content had little influence on the activation behavior. However, increasing the concentration to 37.5 mg mL^−1^ resulted in a noticeable increase in activation strain to over 6%. This increase is attributed to excessive polymer accumulation around the LMPs, which hinders efficient stress transfer to the oxide shell, thereby delaying the onset of activation. The strain-activated MLHMs also exhibited excellent uniformity in electrical properties, enabling their use as reliable interconnects in large-area stretchable electronics (Figs. 4d and S24). To assess the long-term stability and mechanical durability of MLHMs after activation, their electrical performance was monitored over one-week storage period under ambient conditions, and cyclic tensile tests were performed over 1000 cycles at a strain range of 0–100% (Figs. 4e and S25). The MLHMs showed no degradation in electrical performance, demonstrating outstanding durability, reliability, and stability for practical applications.

### MLHMs-Based Circuits and Related Applications

**Fig. 5:**
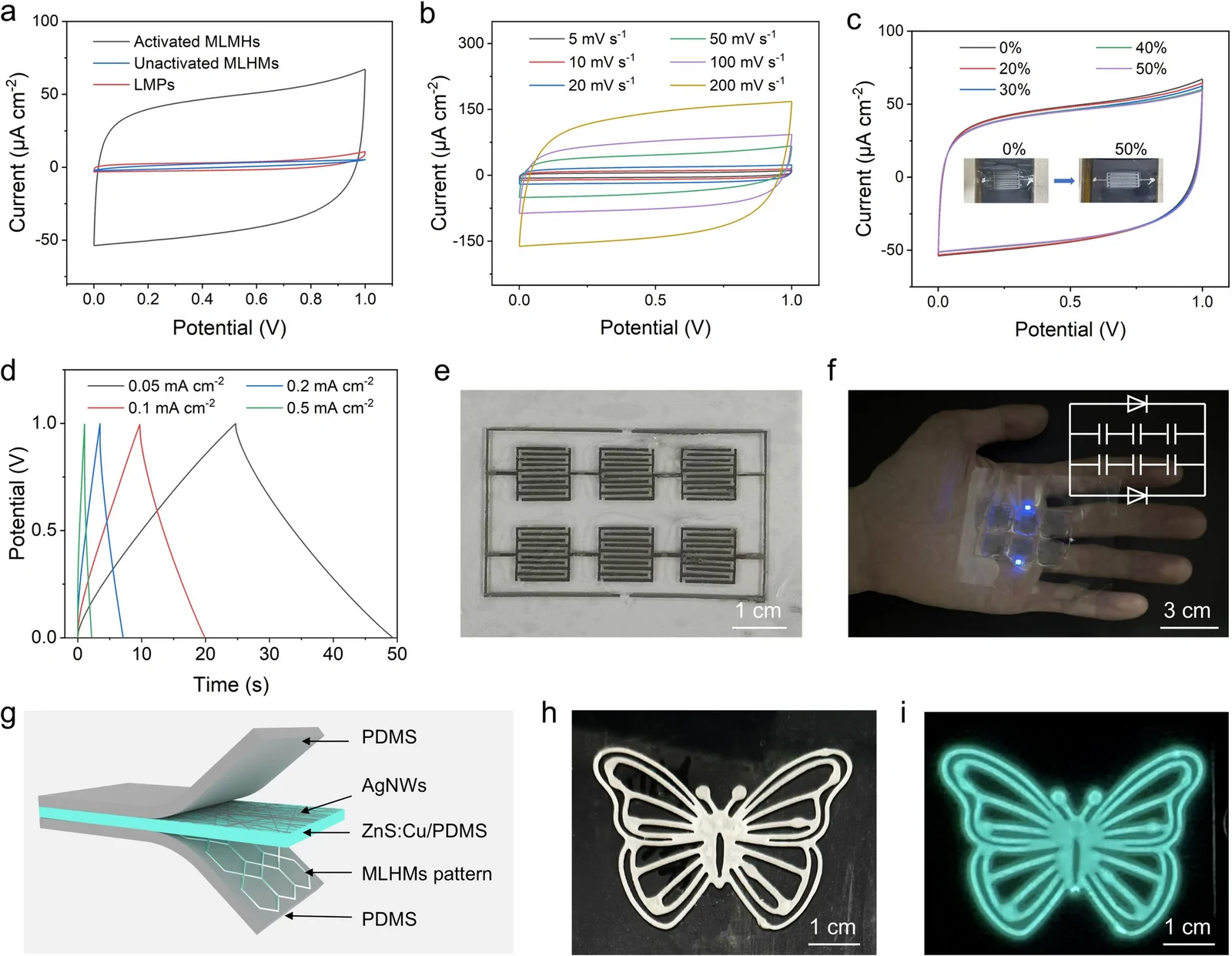
MLHM-based micro-supercapacitors (MSCs) and electroluminescent device. **a** CV curves for MSCs with different electrodes including activated MLHMs, unactivated MLHMs, and LMPs. Measurements were conducted at a scan rate of 50 mV s^−1^ within a voltage window of 0–1 V. **b** CV curves of MSCs based on activated MLHMs at various scan rates. **c** CV curves of MSCs under different mechanical stretching conditions, measured at a scan rate of 50 mV s^−1^. **d** Galvanostatic charge–discharge (GCD) curves of MLHM-based MSCs at different current densities. **e** Photograph of a 2 × 3 MSCs array. **f** Photograph of a MSCs array integrated with two LEDs. **g** Schematic diagram of the structure of the stretchable electroluminescent device, consisting of a patterned MLHMs bottom electrode, a ZnS/PDMS electroluminescent layer, a silver nanowire top electrode and a PDMS encapsulation layer. **h** Butterfly-shaped MLHMs pattern fabricated on a TPU film via 3D printing. **i** Photograph of the electroluminescent device illuminated under an AC field of 2.9 V μm^−1^ at 1 kHz

MLHMs are highly promising to simultaneously achieve high electrochemical activity and mechanical stretchability in energy storage electrodes. To demonstrate their applicability, stretchable all-solid-state micro-supercapacitors (MSCs) were fabricated using MLHM-based electrodes (Fig. S26). Interdigitated MLHM-based electrodes were stencil-printed onto a TPU film, covering an area of 1 × 1 cm^2^, with both line widths and spacings of 500 μm. In these printed electrodes, MXene nanosheets acted as the electrochemically active material, while the activated LM functioned as the current collector. As a control, MSCs with pure LM electrodes showed a low areal capacitance of 0.10 mF cm^−2^ due to the absence of electrochemically active materials. In the unactivated MLHM electrodes, the insulating oxide shells of the LMPs impeded the formation of a continuous liquid metal phase for electron transport. As a result, charge conduction primarily relied on the MXene network, leading to high internal resistance and a notably low areal capacitance of 0.04 mF cm^−2^. In contrast, MSCs with strain-activated MLHM electrodes exhibited quasi-rectangular cyclic voltammetry (CV) curves, which are typical of electric double-layer capacitors. These devices achieved a significantly improved areal capacitance of 1.71 mF cm^−2^, which is 17 times higher than that of MSCs using LM electrodes (Fig. 5a).

The CV curves of MSCs with activated MLHM electrodes were recorded at scan rates ranging from 5 to 200 mV s^−1^ (Fig. 5b). All curves exhibited a quasi-rectangular shape of electric double-layer capacitors. The variation of areal capacitance with scan rate is shown in Fig. S27a, revealing a gradual decrease as the scan rate increased, which is attributed to limited ion diffusion at higher rates. Figure 5c presents the CV curves of MSCs under uniaxial tensile strain from 0 to 50%. A slight decrease in capacitance was observed with increasing strain. The initial areal capacitance of 1.71 mF cm^−2^ decreased to 1.62 mF cm^−2^ at 50% strain, corresponding to a high capacitance retention of 94.7%. Meanwhile, the ESR decreased from 181.3 to 136.3 Ω as the tensile strain increased. This decrease in ESR is likely associated with enhanced interfacial contact between the electrode and ionogel under tensile deformation (Fig. S27b). This result demonstrates the excellent mechanical robustness and electrochemical stability of MLHMs electrodes under stretching. Galvanostatic charge–discharge (GCD) measurements were conducted at various current densities (Fig. 5d). The nearly symmetric triangular shapes of the GCD curves indicate ideal capacitive behavior and good charge–discharge reversibility. The ESR initially increased during the early cycling stage and subsequently stabilized, showing an overall increase of ~ 10.6% after 5000 cycles. Meanwhile, the MSCs retained 84.1% of their initial capacitance after 5000 charge–discharge cycles, demonstrating moderate cycling stability (Fig. S27c, d). Our MSCs exhibit comparable cycling performance and good tensile adaptability, highlighting their advantage over conventional rigid MXene-based MSCs (Table S3). To demonstrate the potential of MLHM-based MSCs as power sources for soft electronic systems, a 2 × 3 MSCs array (three cells connected in series and two in parallel) was designed to meet the voltage requirements of light-emitting diodes (LEDs) (Fig. 5e). Six MLHM interdigitated electrodes and their interconnections were stencil-printed onto a TPU substrate. Ion gels were then applied to the interdigitated electrode regions to complete the MSC array fabrication. The resulting MSCs array had an area of 15 cm^2^ (3 cm × 5 cm). Before use, a slight strain was applied to activate the electrode conductivity, and the MSCs array were charged at 3 V. When connected with LEDs, the MSCs array successfully powered them (Fig. 5f), producing bright illumination and demonstrating its promise as reliable energy storage components for flexible electronic systems.

In addition to serving as electrode materials for energy storage, MLHMs were further explored as functional electrodes in light-emitting device. ACEL devices operate by applying an alternating electric field across the luminescent layer, where light emission is induced through field-driven excitation processes. The electrode layer is essential for maintaining a stable and uniform electric field. Patterned MLHM electrodes were fabricated via 3D printing and integrated into alternating current electroluminescent (ACEL) devices. A fully stretchable ACEL device with a typical sandwich architecture was constructed (Fig. 5g). The bottom electrode, featuring a butterfly-shaped pattern, was printed directly onto a PDMS substrate using MLHM ink (Fig. 5h). The emissive layer was composed of ZnS:Cu phosphors dispersed in a PDMS matrix, while silver nanowires (AgNWs) were spray-coated as the transparent top electrode (Fig. S28a). The device exhibited stable blue light emission under an AC filed of 2.9 V μm^−1^ at 1 kHz (Fig. 5i), and maintained normal operation under a 20% uniaxial strain, demonstrating the excellent mechanical robustness and multifunctionality of MLHMs for use in stretchable optoelectronic applications (Fig. S28b and Video S3).

**Fig. 6:**
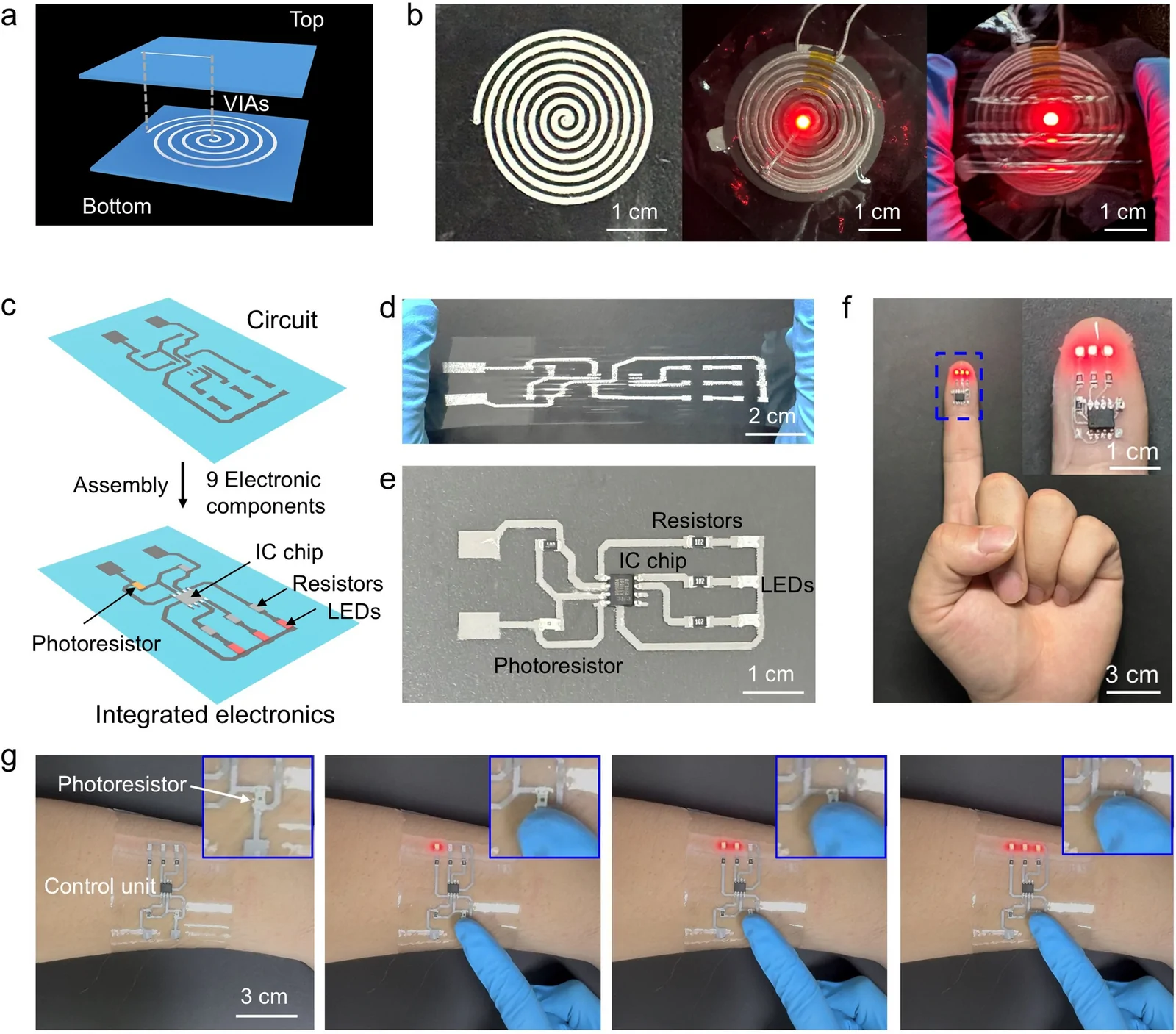
MLHM-based functional circuit. **a** Schematic illustration of a double-layer structural layout with VIAs for installing an LED in a wireless device. **b** Optical image of a 6-turn coil fabricated by 3D printing MLHMs onto a TPU substrate, with both line width and spacing of 1 mm (left), the wireless device powering a LED (middle), and under stretching state (right). A commercial 5 V coil is used as energy transmission source. **c** Schematic diagram of circuit lines and electronic components assembled with MLHMs for flexible interactive display system. Photographs of MLHM-based circuits** d** printed on a stretchable TPU film, **e** integrated with electronic components, and** f** a miniaturized version on the fingertip. **g** Photographs of a flexible MLHM-based interactive display system mounted on the forearm. When the finger approaches the photoresistor, the reduced light intensity sequentially triggers multiple LEDs to turn on

The applicability of MLHMs was further demonstrated in stretchable wireless power transfer systems. A dual-layer coil structure incorporating LM vertical interconnect accesses (VIAs) was designed to integrate a micro-LED as the load (Figs. 6a and S29a). A six-turn receiver coil was stencil-printed onto a TPU substrate, with a line width and spacing of 1 mm (Fig. 6b). For wireless energy transmission via inductive coupling, a commercially available 5 V wireless charger equipped with a copper transmitting coil was used as the power source. When the MLHM-based receiver coil was aligned and placed on top of the transmitting coil, it effectively harvested electromagnetic energy to power the micro-LED. Importantly, the wireless power transmission remained stable under mechanical deformation of the stretchable device (Fig. 6b). Moreover, this inductive wireless power transfer system was capable of transmitting energy through various barriers, such as glass and water, highlighting the excellent robustness and versatility of the MLHM-based system (Fig. S29b).

Flexible printed circuit boards (F-PCBs) not only serve as platforms for electrical interconnection but also provide integration bases for sensors, microcontrollers, and energy storage units, thereby enabling the development of highly integrated stretchable systems such as flexible batteries, wearable health monitors, and interactive wearable displays. Given their patternability and stretchability, MLHMs are well-suited to serve as stretchable circuits integrated into F-PCBs. To validate this application, we fabricated F-PCBs by printing MLHM-based circuits onto flexible substrates and assembling them with commercial electronic components (Fig. S30a). As a proof of concept, the circuits featuring letters "LMP" were printed onto a TPU substrate, and 26 micro-LEDs were installed to form a stretchable display array. The display operated reliably at a low driving voltage of 3 V (Fig. S30b). Even under various mechanical deformations such as bending, twisting, and stretching, the LED array maintained stable brightness without noticeable degradation (Fig. S30c**–**f and Video S4), confirming the robust electrical conductivity and mechanical resilience of the MLHM circuits.

As a proof of concept for interactive wearable electronics, a stretchable light-sensitive display circuit was designed and fabricated using MLHMs (Fig. 6c, d). The circuit was integrated with a commercial IC chip and discrete electronic components, including a photoresistor, resistors, and LEDs, to construct a F-PCB (Fig. 6e). The resulting compact circuit, with a size of ~ 1 cm × 2 cm, was small enough to be securely worn on the index finger (Fig. 6f). This integrated electronic system enabled non-contact interactive display functionality; for example, when worn on the forearm under ambient lighting, none of the LEDs were illuminated. As the user’s finger gradually approached the photoresistor, decreasing the incident light intensity, the LEDs turned on sequentially (Fig. 6g). The touchless electronic system allows for intuitive and hygienic interaction, making it particularly promising for future smart wearables, gesture-controlled electronics, and human–machine interfaces.

## Conclusions

In summary, we present a multifunctional MLHMs platform, where MXene nanosheets are assembled around LMPs via coordination interactions. These hybrid microparticles can be directly printed onto diverse substrates with strong adhesion. The robust interfacial bonding between the rigid MXene layers and the oxide shell of the LMPs enables efficient stress transfer under deformation, triggering the rupture of the oxide layer and the formation of continuous conductive pathways at a minimal strain of just 2.5%. As a result, the printed MLHM patterns exhibit a high electrical conductivity of 3.7 × 10^5^ S m^−1^ and outstanding stretchability of ~ 700%. By combining the superior conductivity of liquid metals with the electrochemical activity of MXenes, MLHMs deliver a unique integration of electrical, mechanical, and electrochemical performance. We further demonstrate the versatility of MLHMs by integrating them into a range of printed electronic devices, including stretchable antennas, electroluminescent devices, and micro-supercapacitors. As a proof of concept, we further developed MLHM-based F-PCBs integrated with a light-sensitive circuit for touchless interactive display. This work highlights MLHMs as a versatile and high-performance material platform for next-generation soft electronics in wearable device, soft robotics, and human–machine interfaces.

## Supplementary Information

Below is the link to the electronic supplementary material.

Supplementary file1 (MP4 7726 kb)

Supplementary file2 (MP4 1314 kb)

Supplementary file3 (MP4 1952 kb)

Supplementary file4 (MP4 2828 kb)

Supplementary file5 (DOCX 13626 kb)
