# Supplementary material for: MXene-Assembled Liquid Metal Hybrid Microparticles for Multifunctional and Stretchable Printed Electronics
Source: Nanomicro Lett. 2026 Apr 3;18:314. doi: 10.1007/s40820-026-02154-3 (PMC13049131; doi:10.1007/s40820-026-02154-3)
Supplement: Supplementary file 5 — Supplementary file5 (DOCX 13626 kb) [file 40820_2026_2154_MOESM5_ESM.docx]

Supporting Information for

**MXene-Assembled Liquid Metal Hybrid Microparticles for** **Multifunctional and Stretchable Printed Electronics**

Rouhui Yu^1^, Jiexin Qiu^1^, Hui Zhu^1^, Xiangheng Du^1^, Jiale Sun^1^, Zishuo Zhang^1^, Long Chen^1^, Zhongyao Fan^1^, Huifang Chen^1^, Meifang Zhu^1^, and Shaowu Pan^1^*

^1^State Key Laboratory of Advanced Fiber Materials, College of Materials Science and Engineering, Donghua University, Shanghai 201620, P. R. China

*Corresponding author. E-mail: [pansw@dhu.edu.cn](mailto:pansw@dhu.edu.cn) (Shaowu Pan)

**Supplementary Figures and Tables**

**
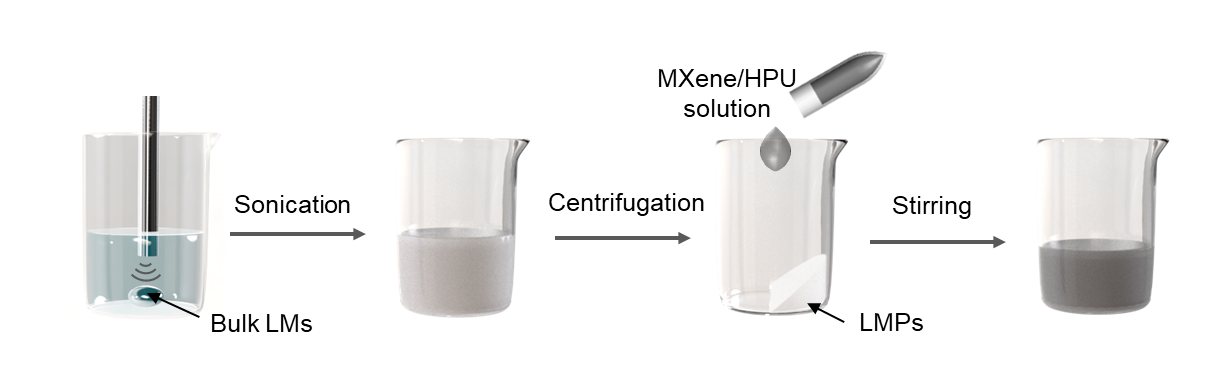
**

**Fig. S1** Schematic illustration of the fabrication of MLHMs-based ink


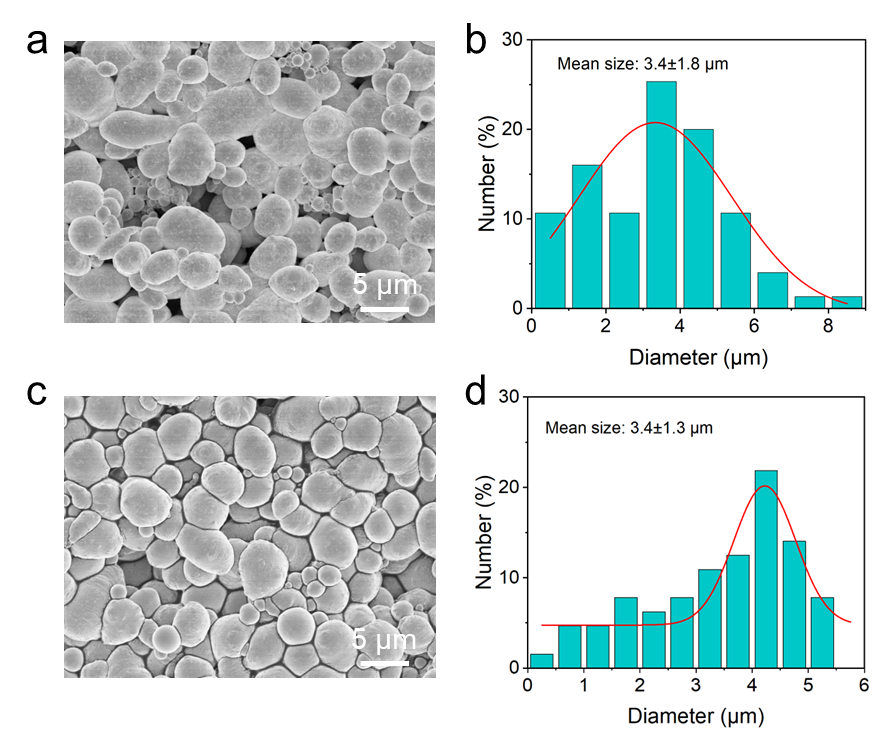


**Fig. S2** **a, c** SEM images of LMPs prepared in ethanol using tip sonication at 300 W for 3 min, without HPU (a) and with HPU (c). **b, d** The plots of particle size distribution of LMPs corresponding to (a) and (c), respectively


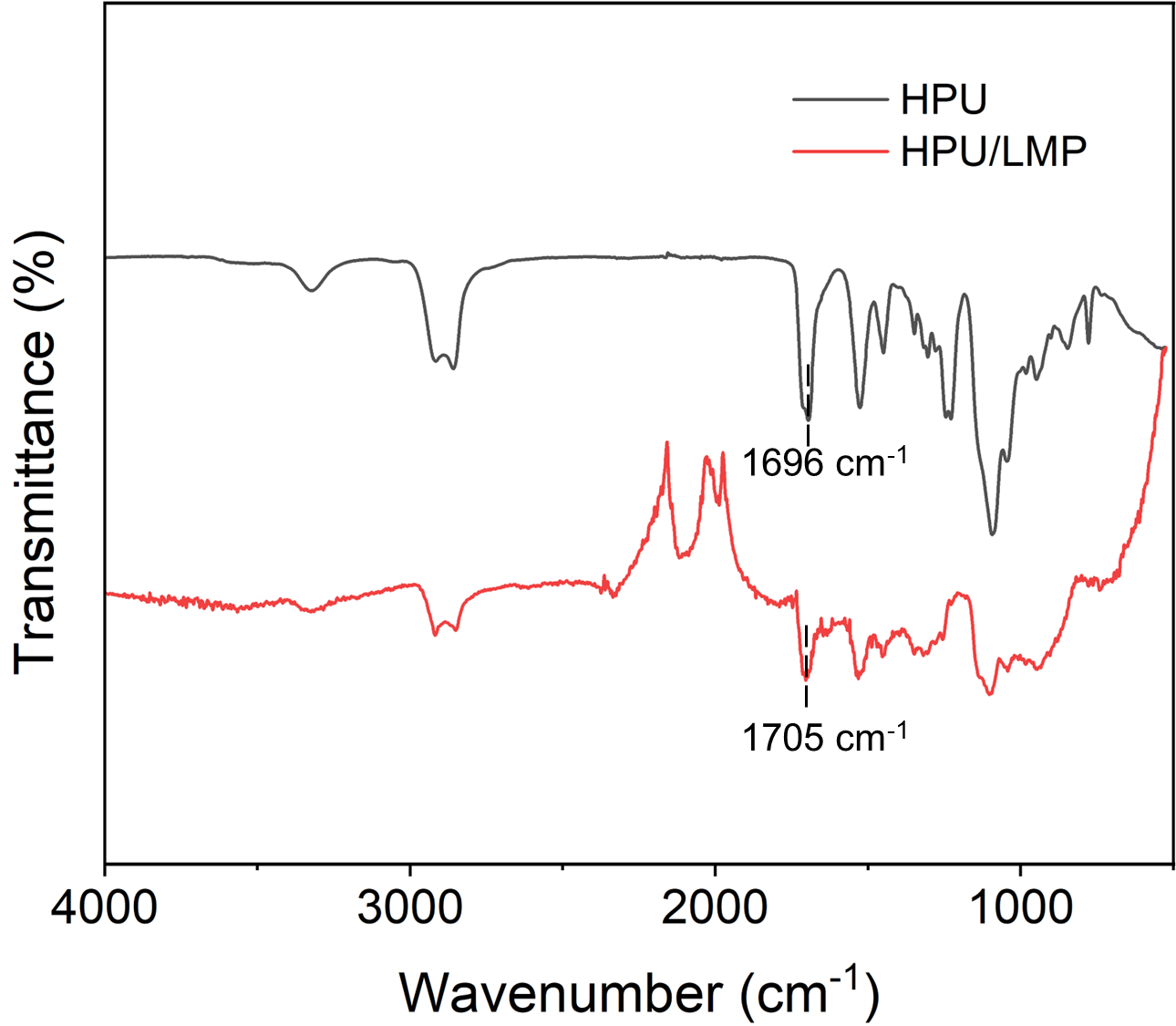


**Fig. S3** FTIR spectrum of HPU and HPU/LMP

**
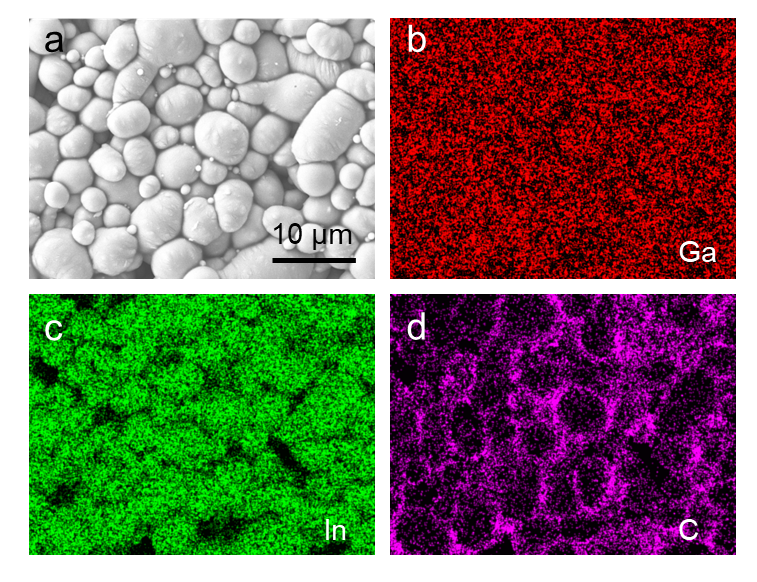
**

**Fig. S4** SEM image and corresponding element mapping images of LMPs/HPU composite


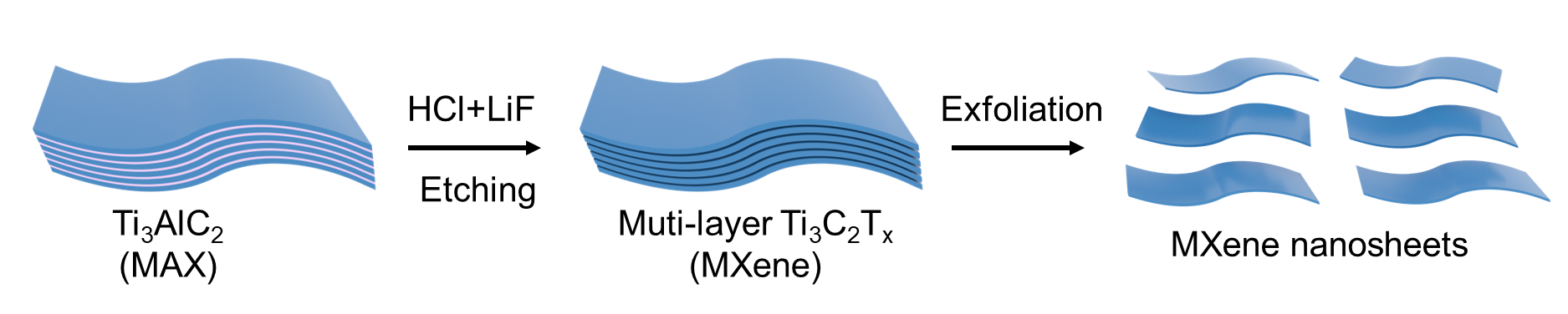


**Fig. S5** Schematic diagram for the fabrication of MXene nanosheets


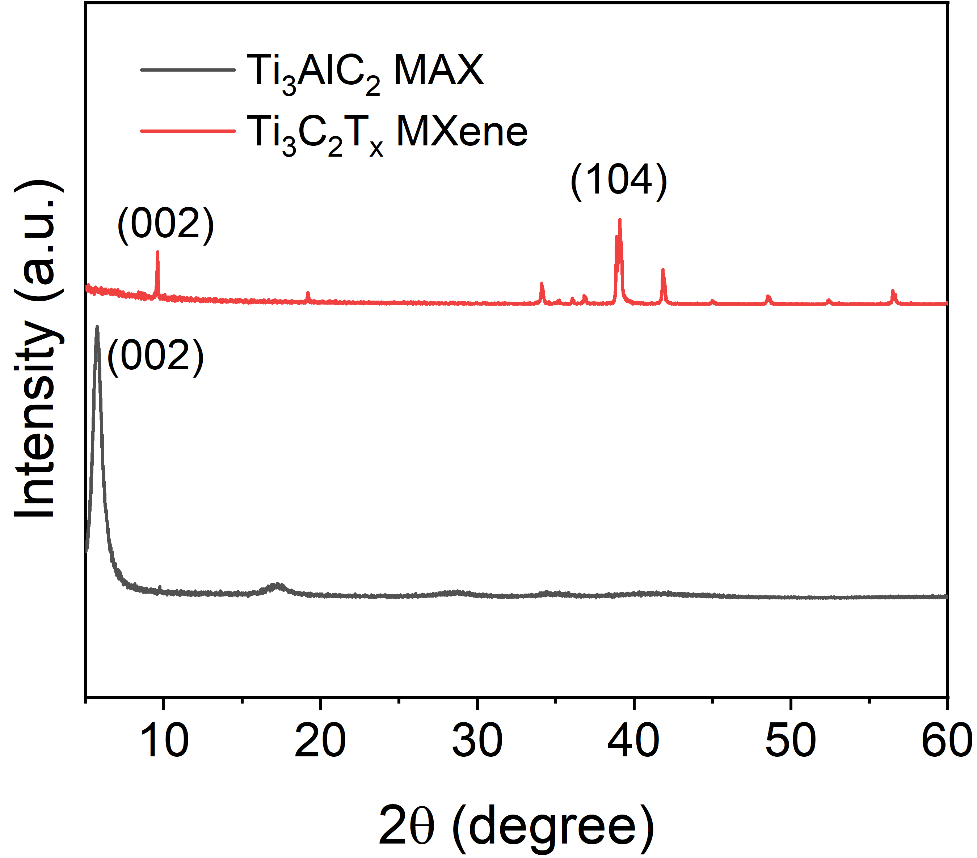


**Fig. S6** X-ray diffraction (XRD) patterns of Ti_3_AlC_2_ MAX and Ti_3_C_2_T_x_ Mxene

**
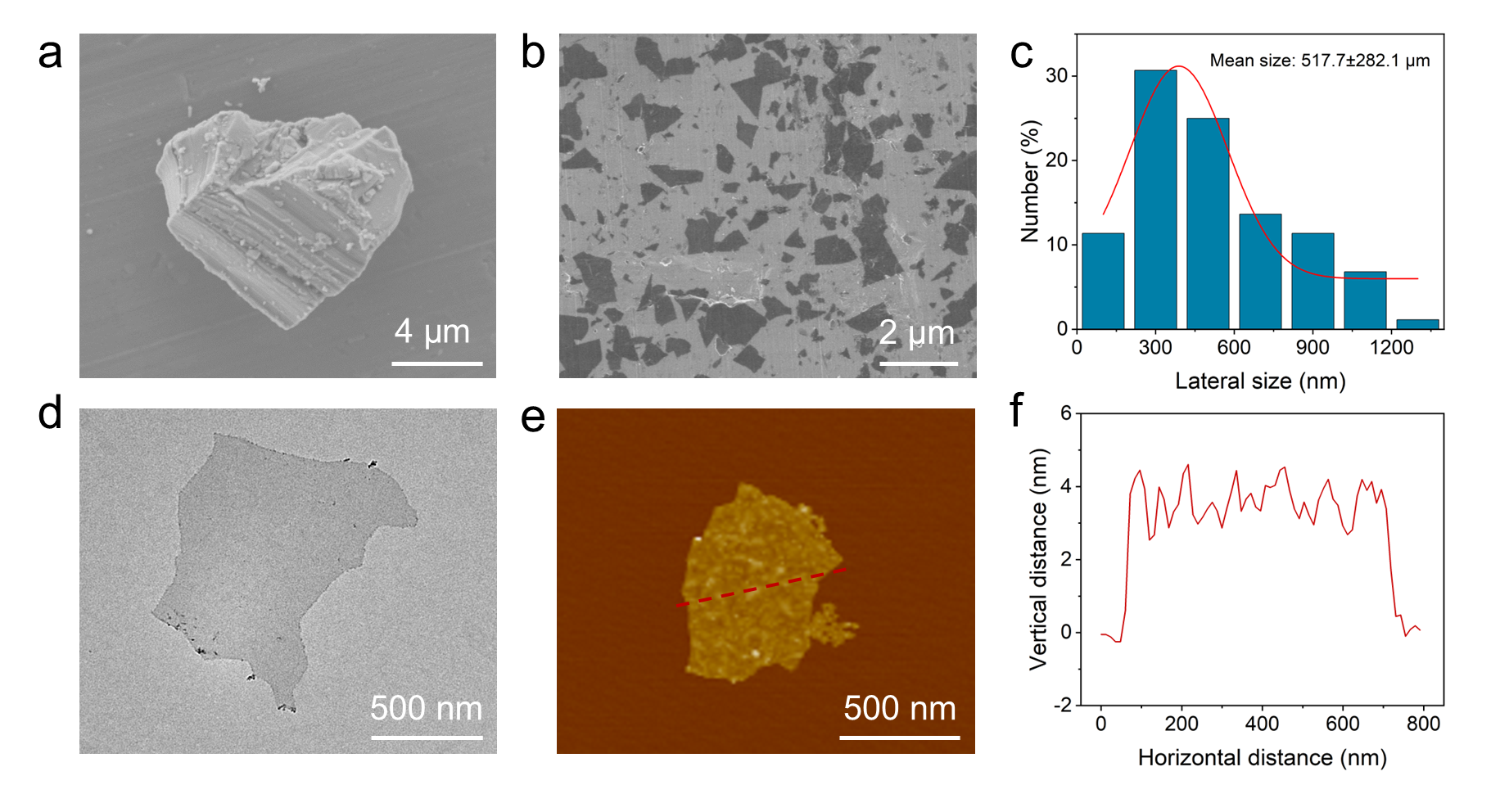
**

**Fig. S7** **a** SEM image of Ti_3_AlC_2_ MAX. **b** SEM image of MXene nanosheets. **c** Size distribution of MXene in (b). **d** TEM image of MXene nanosheet. **e** AFM image of MXene nanosheet. **f** Vertical distance of the MXene nanosheet in (e); the thickness of the MXene nanosheet is ~3 nm

**
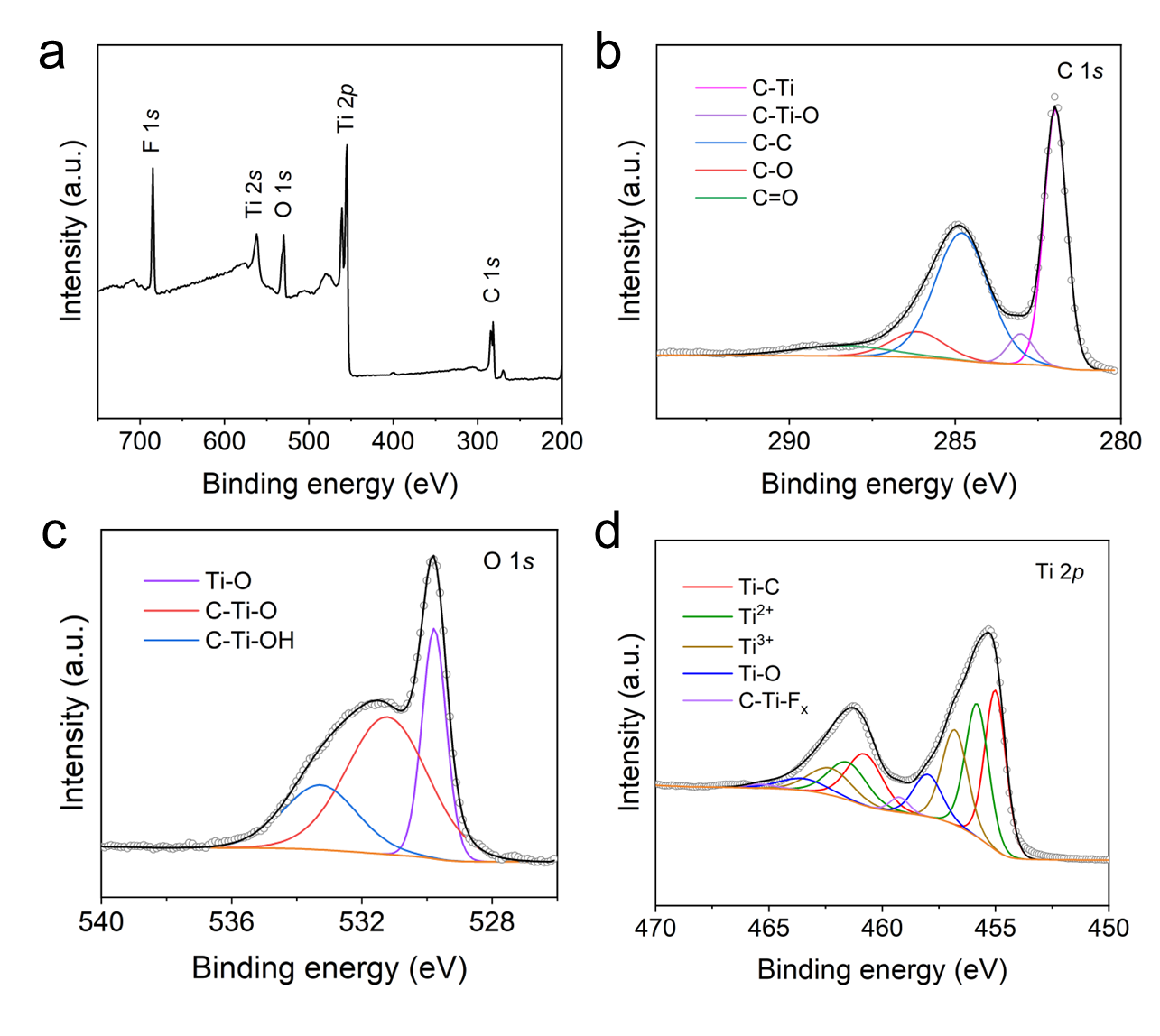
**

**Fig. S8 a** XPS spectrum of MXene nanosheets. The high resolution XPS spectra for C 1*s* (**b**), O 1*s* (**c**), and Ti 2*p* (**d**)


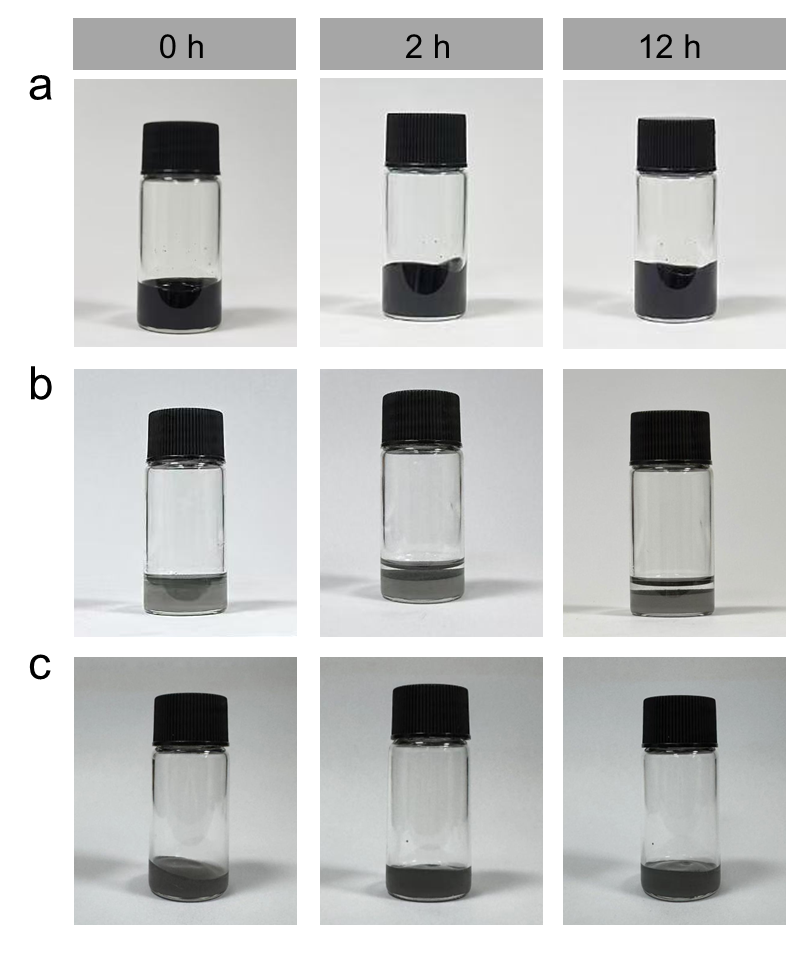


**Fig. S9** Images of ethanol-water solution containing MXene (**a**), MXene/LMPs (**b**), and MXene/LMPs/HPU (**c**). The samples were stored under ambient conditions over 12 h


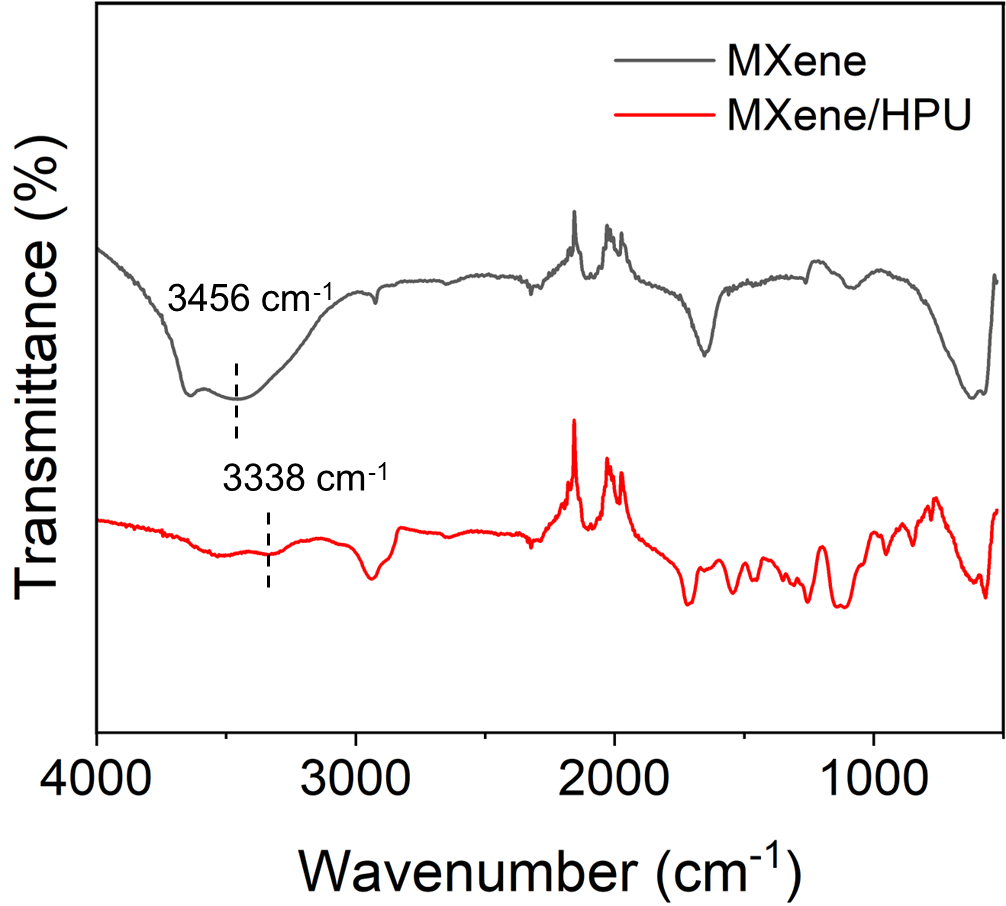


**Fig. S10** FTIR spectrum of MXene nanosheets and MXene/HPU


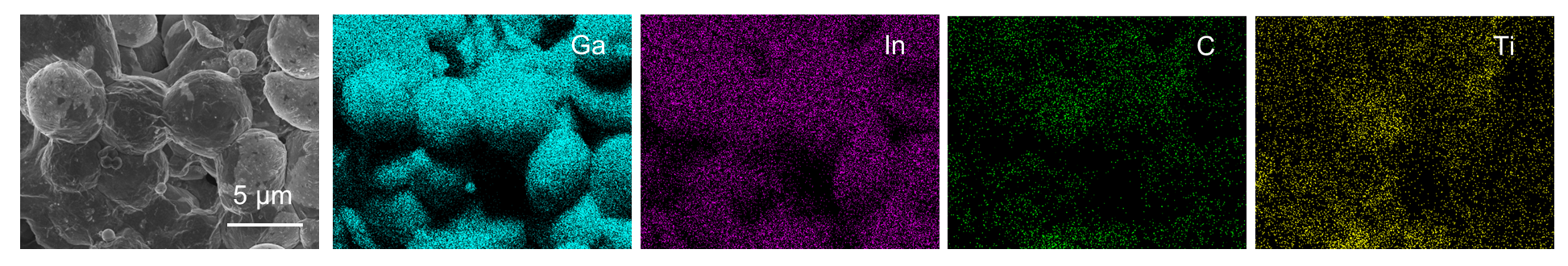


**Fig. S11** SEM images of the MLHMs after washing, along with elemental mapping of Ga, In, C, Ti. The washing was performed using a 95% ethanol and 5% water solution with shaking for 3 minutes


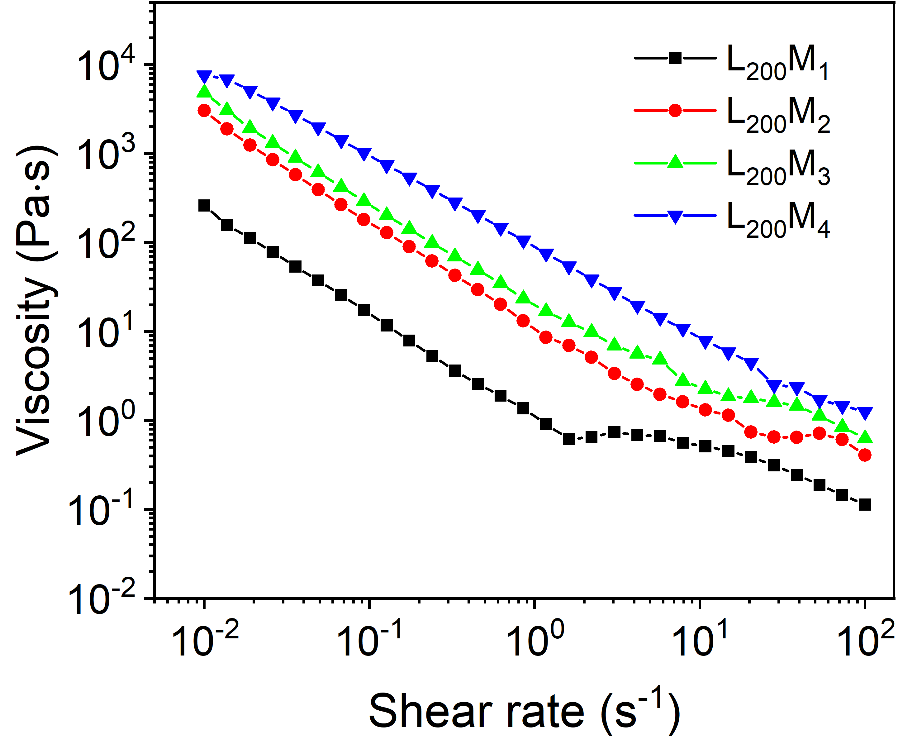


**Fig. S12** Viscosity of MLHMs-based ink as a function of shear rate for different mass ratios of LMPs to MXene. L_x_M_y_ represents a mass ratio of x: y between LMPs and MXene


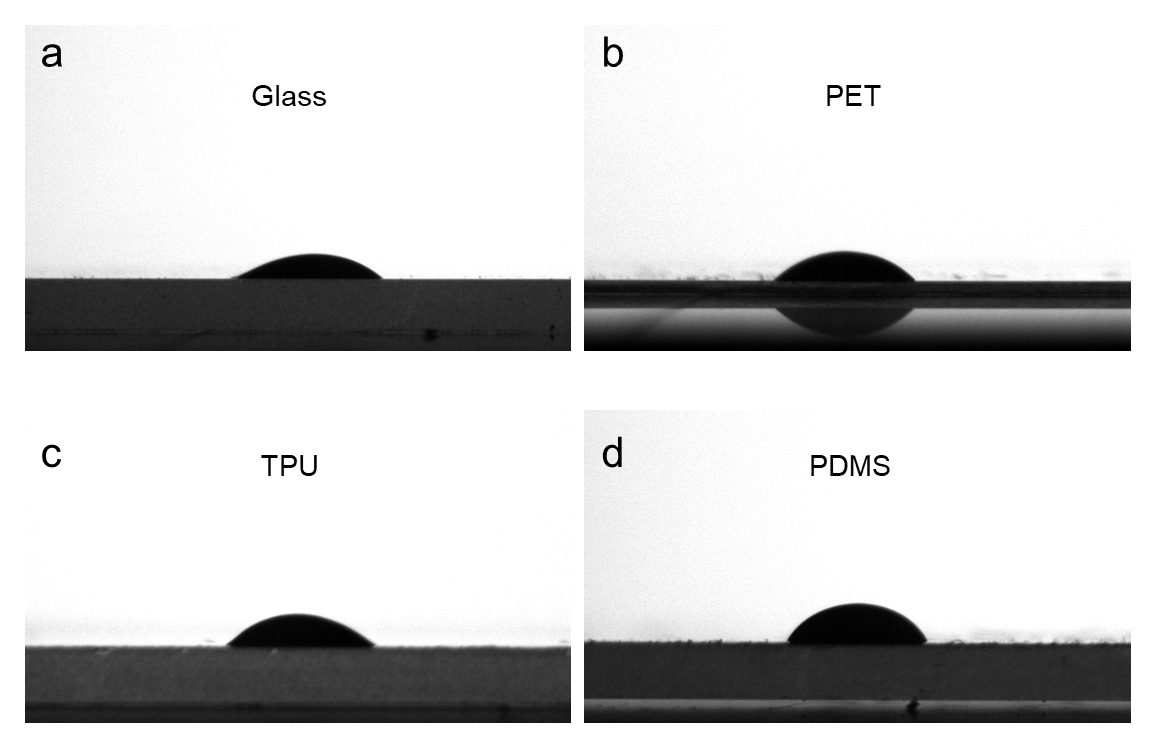


**Fig. S13** Contact angle images of the MLHMs-based ink on different substrates, including glass, PET, TPU, and NH_2_-functionalized PDMS


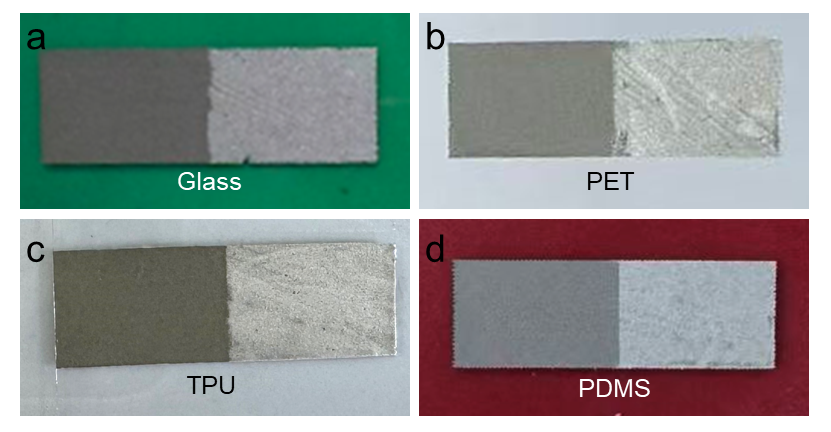


**Fig. S14** Photographs of the tape peel test for MLHMs on different substrates, including glass, PET, TPU, and NH_2_-functionalized PDMS. The left side of each image shows the pristine state, and the right side shows the state after peeling. After peeling, a continuous MLHMs layer remains adhered to the substrates, indicating stable interfacial adhesion


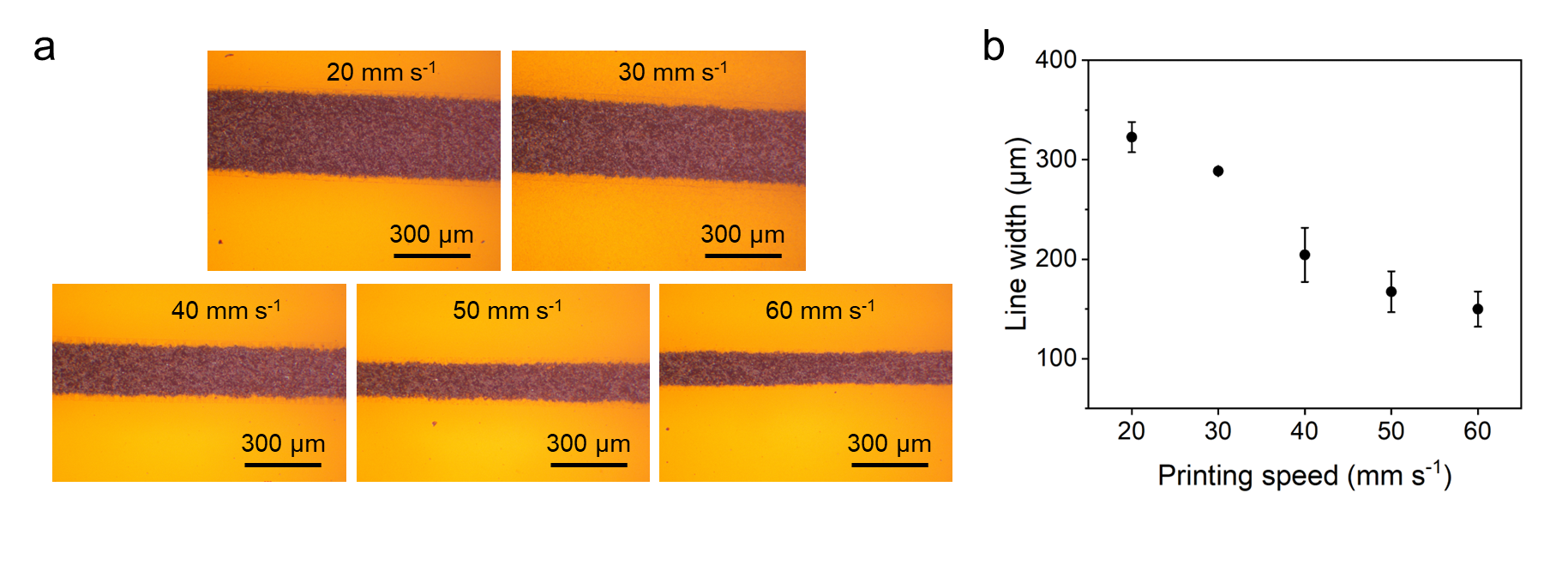


**Fig. S15 a** Optical images of printed lines at different printing speeds. **b** The corresponding linewidths were obtained using a 30 G nozzle (inner diameter: 160 μm) under an extrusion pressure of 15 kPa


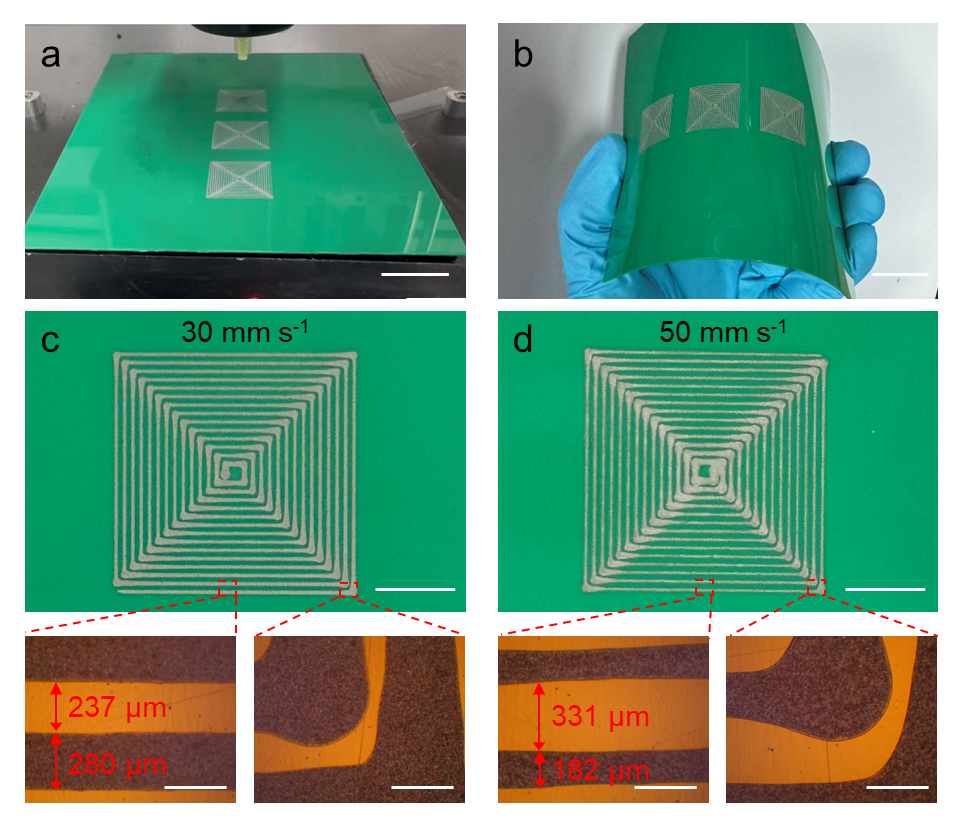


**Fig. S16 a** Optical image of printed patterns across multiple regions on a PET substrate (scale bar: 1.5 cm. The MLHMs ink contains 37.5 mg mL^-1^ of HPU. **b** Optical image of printed patterns under bending deformation (scale bar: 1.5 cm). **c** Optical image of a printed pattern with localized enlarged views obtained at an extrusion speed of 30 mm s^-1^ (scale bar: 0.5 cm; enlarged views: 300 μm). **d** Optical image of a printed pattern with localized enlarged views obtained at an extrusion speed of 50 mm s^-1^ (scale bar: 0.5 cm; enlarged views: 300 μm)

At an extrusion speed of 30 mm s^-1^, the printed lines exhibit a linewidth of ~280 μm with an interline spacing of ~237 μm. When the extrusion speed is increased to 50 mm s^-1^, the linewidth decreases to ~182 μm, while the spacing increases to ~331 μm. The linewidth broadening and corner rounding are observed at turning points, which can be attributed to transient velocity reduction and local material accumulation during directional changes. Importantly, the printed traces remain continuous and well-defined without observable edge fracture or delamination, indicating stable deposition behavior.


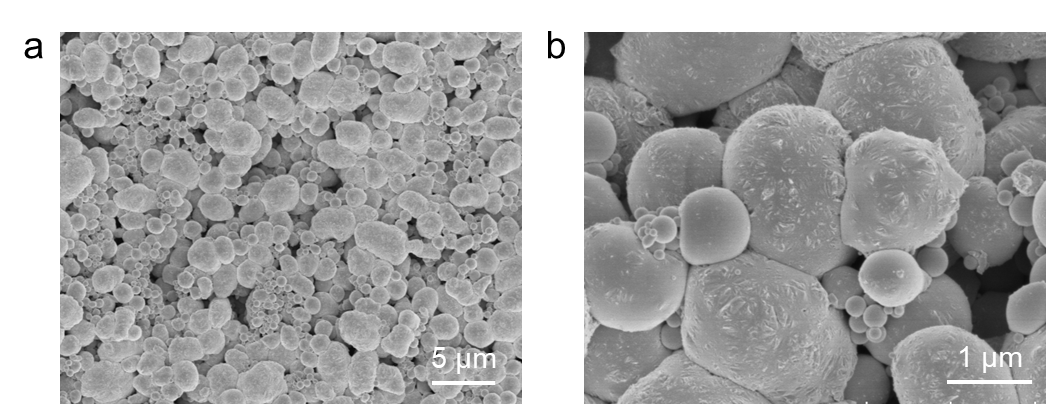


**Fig. S17** SEM images of pure LMPs after solvent evaporation


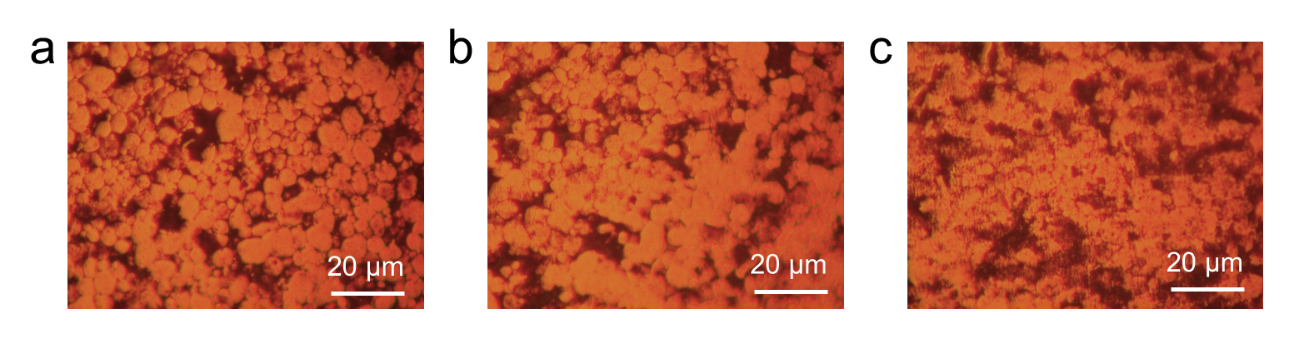


**Fig. S18** Optical images of the backside of TPU films printed with the MLHMs-based ink. **a** Initial state. **b** After 10 cycles of 5% tensile strain. **c** After 10 cycles of 100% tensile strain

**
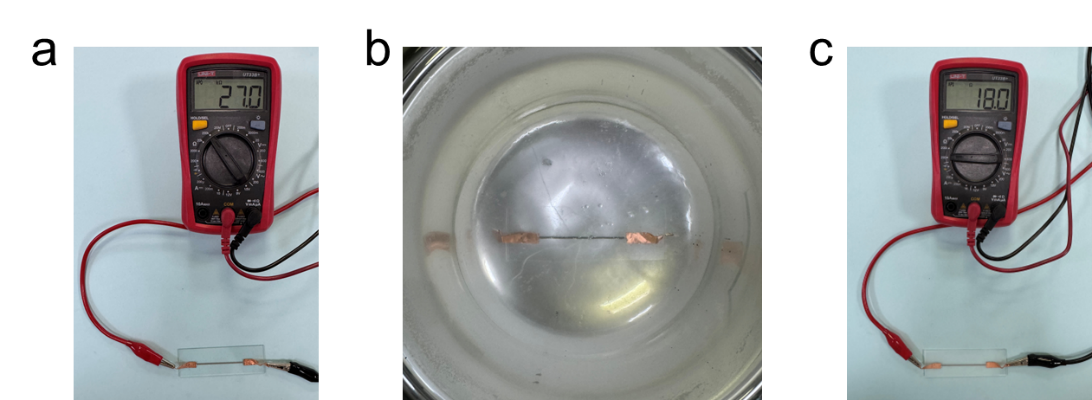
**

**Fig. S19** **a-c** Optical photographs showing (a) the initial resistance of a line printed on a glass substrate using MLHMs-based ink, (b) the sample immersed in liquid nitrogen, and (c) the resistance of the printed MLHMs line after immersion


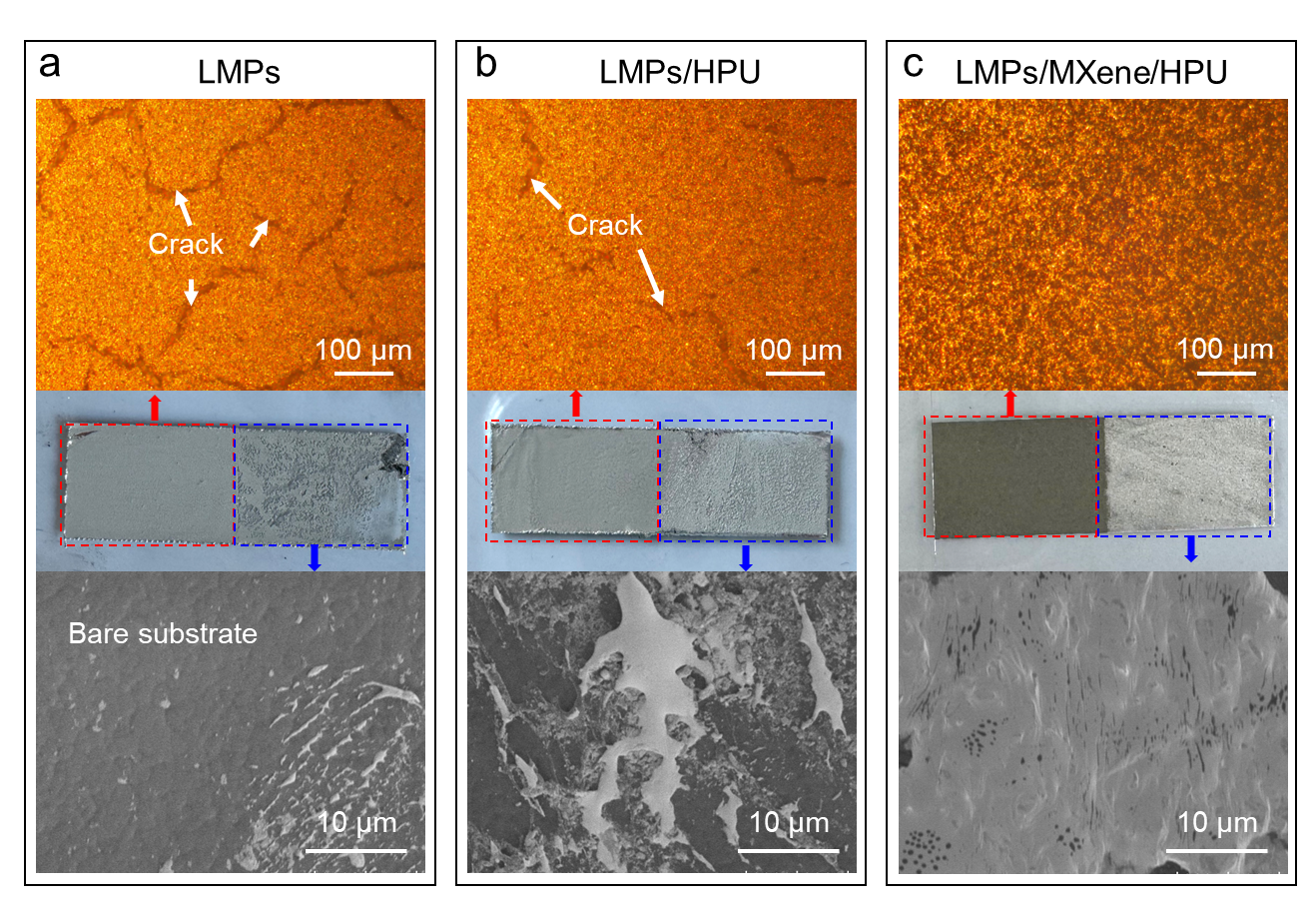


**Fig. S20 a-c** Optical and SEM images of (a) LMPs, (b) LMPs/HPU, and (c) LMPs/MXene/HPU composites (MLHMs). The red dashed box indicates the surface before the tape adhesion test, and the blue dashed box indicates the surface after tape removal


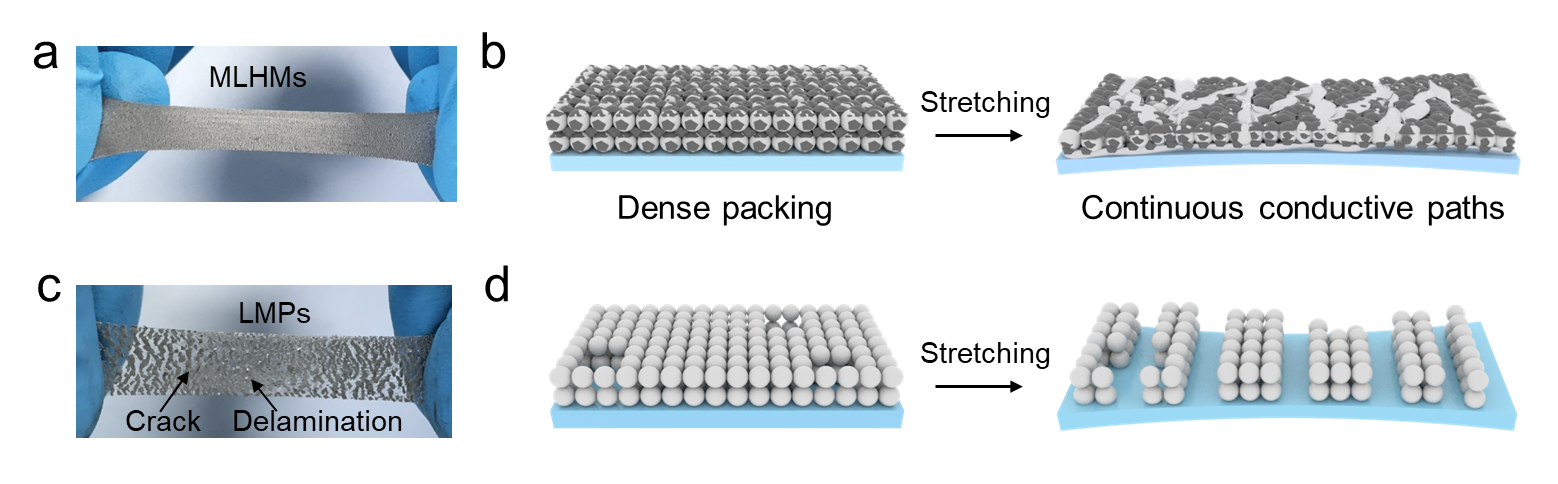


**Fig. S21 a** Optical photograph of the MLHMs-coated TPU film under a tensile strain of 50%. **b** Schematic illustration of the MLHMs-coated film before and after stretching. **c** Optical photograph of the LMPs-coated TPU film under a tensile strain of 50%. **d** Schematic illustration of the LMPs-coated film before and after stretching

**
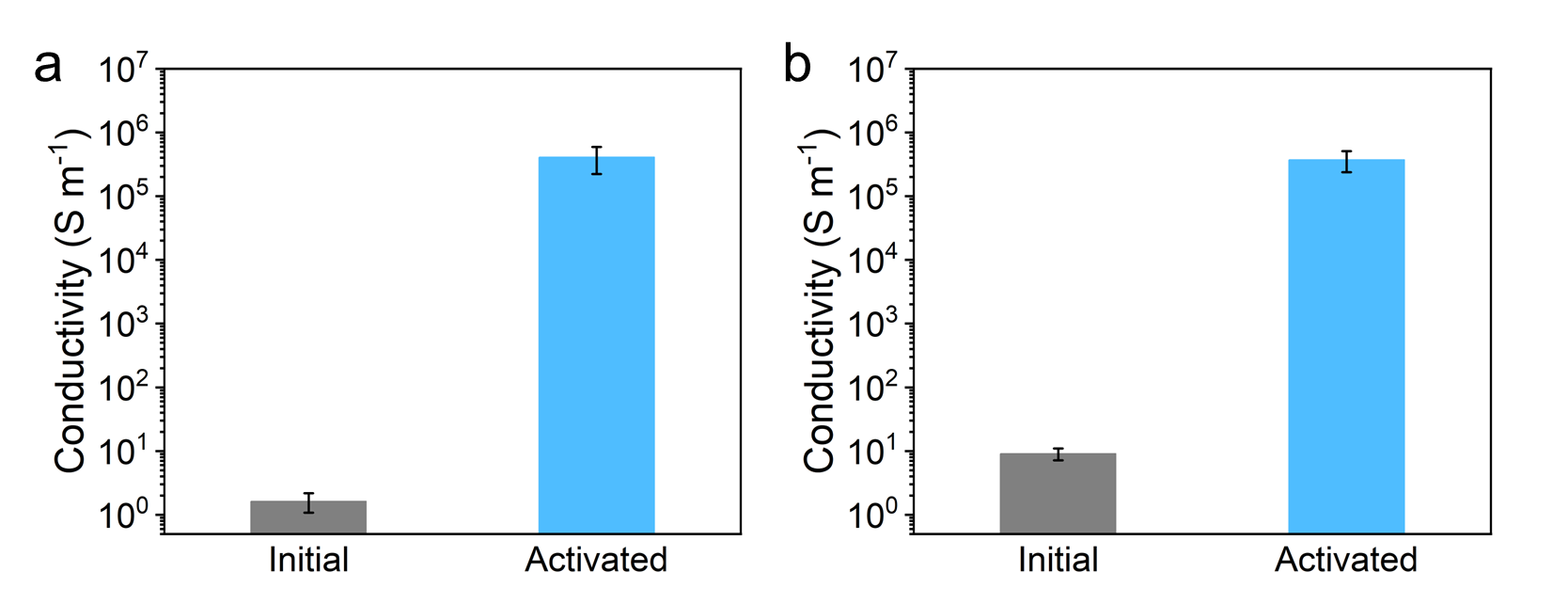
**

**Fig. S22** **a, b** Conductivity of (a) M_1.5_L_100_HMs, and (b) M_2_L_100_HMs before and after activation. Data are presented as mean values ± s.d. (n = 5 independent samples)


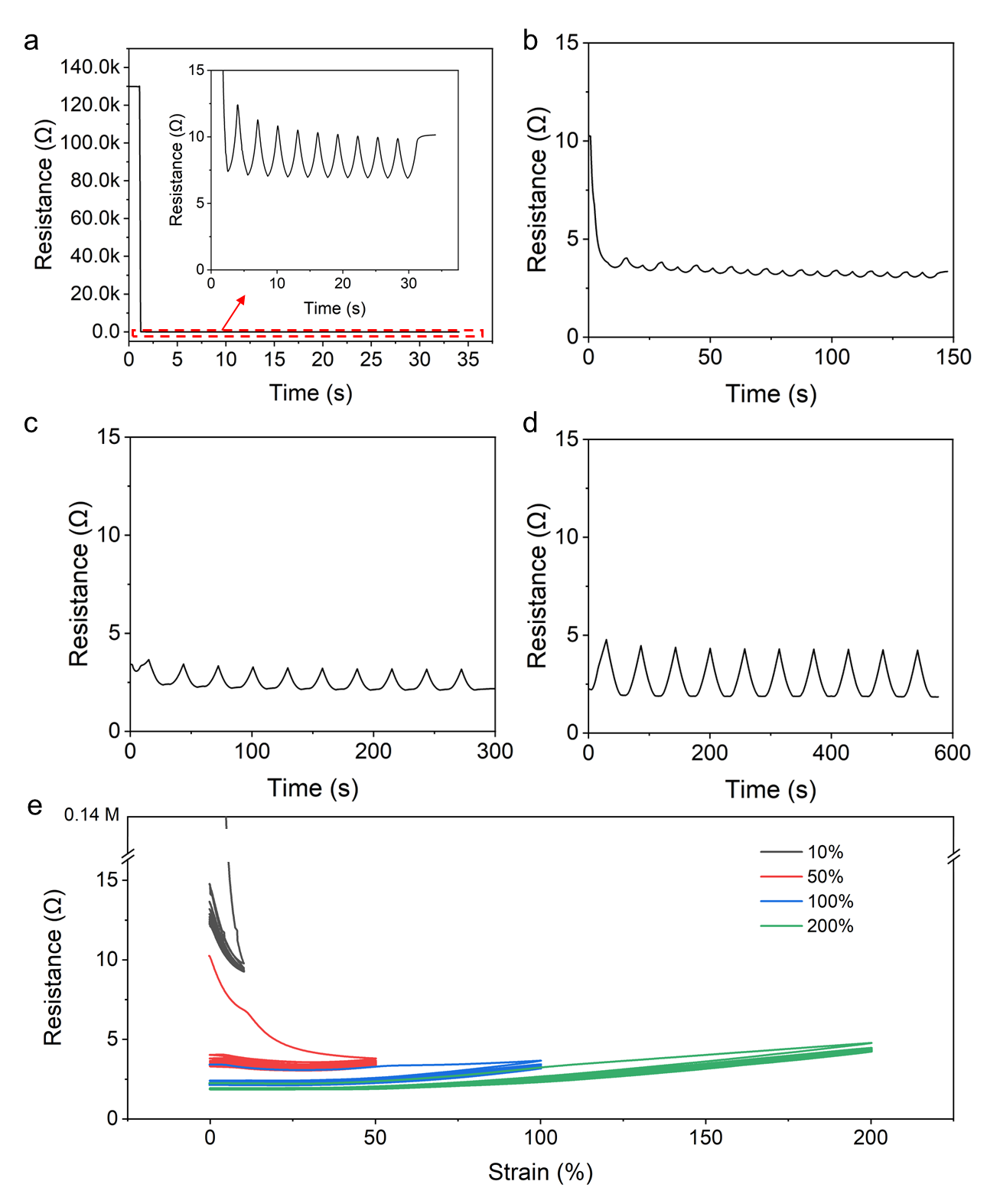


**Fig. S23 Electromechanical performance under cyclic tensile strain.** **a**–**d** Resistance–time responses of the device under cyclic tensile strains of 10% (a), 50% (b), 100% (c), and 200% (d), respectively, each measured for 10 loading–unloading cycles. (e) Resistance as a function of applied strain during 10 cycles at 10%, 50%, 100%, and 200% strain


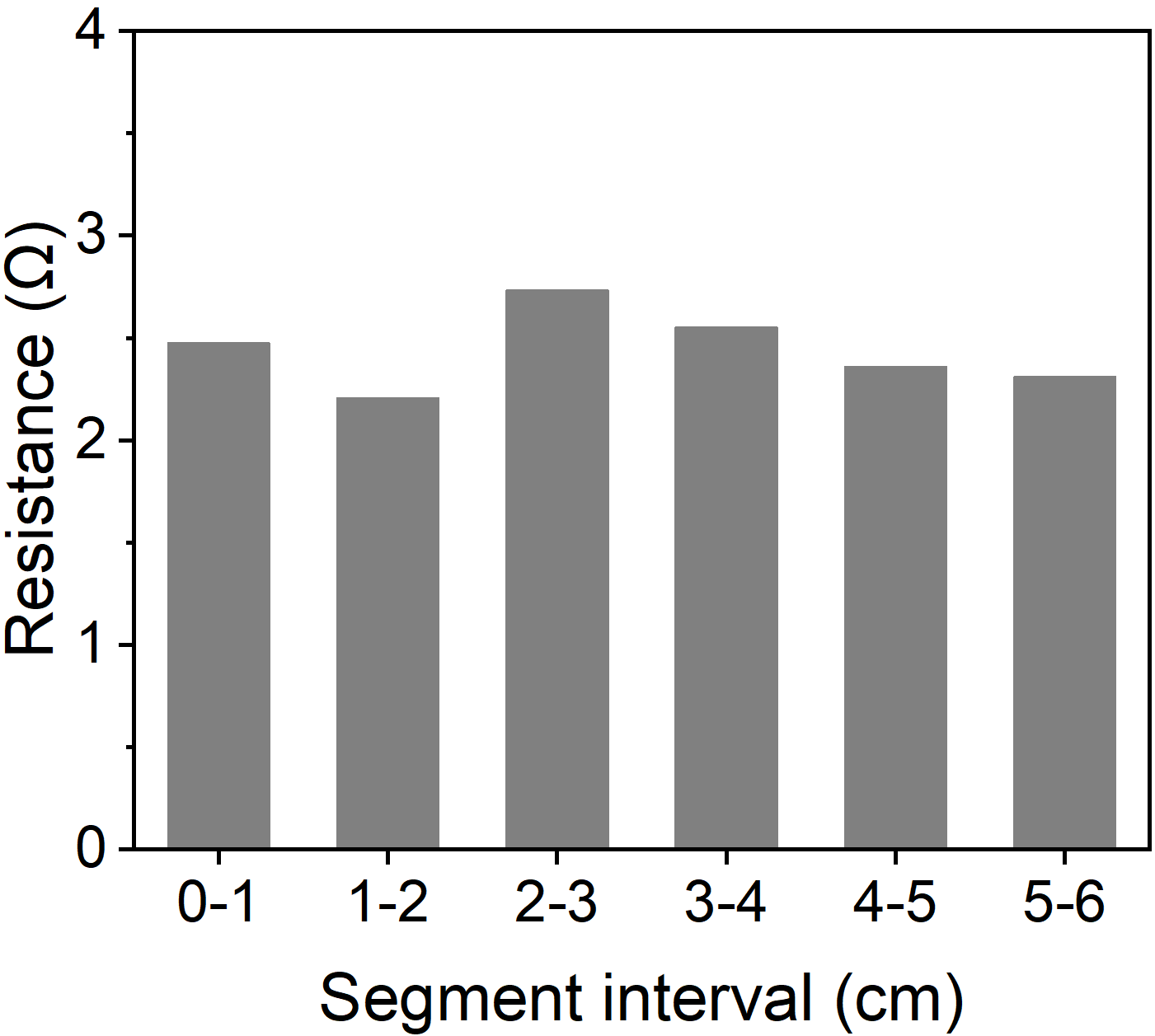


**Fig. S24** Spatial resistance of the activated MLHMs corresponding to Figure 4d. The average resistance of individual 1 cm segments was 2.49 Ω with a standard deviation of 0.24 Ω (coefficient of variation, CV ≈ 9.4%)


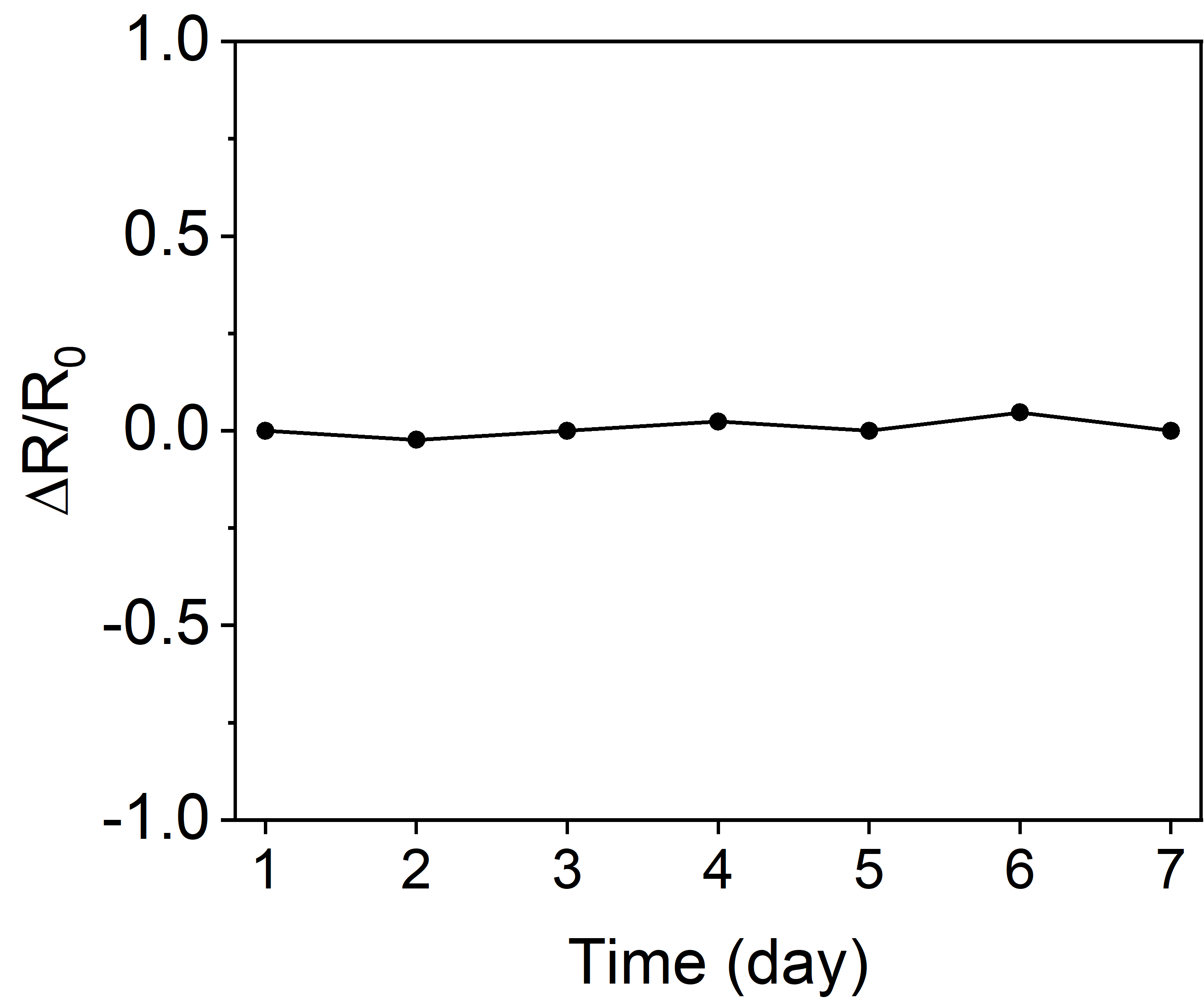


**Fig. S25** Relative resistance change of the activated MLHM over time


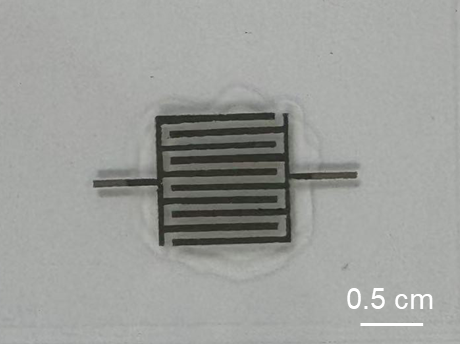


**Fig. S26** Photograph of all-solid-state MSCs fabricated by stencil-printing MLHMs-based interdigitated electrodes on a TPU substrate, followed by coating with an ionic gel electrolyte. The electrode area is 1 cm^2^, with a line width and inter-electrode gap of 0.5 mm


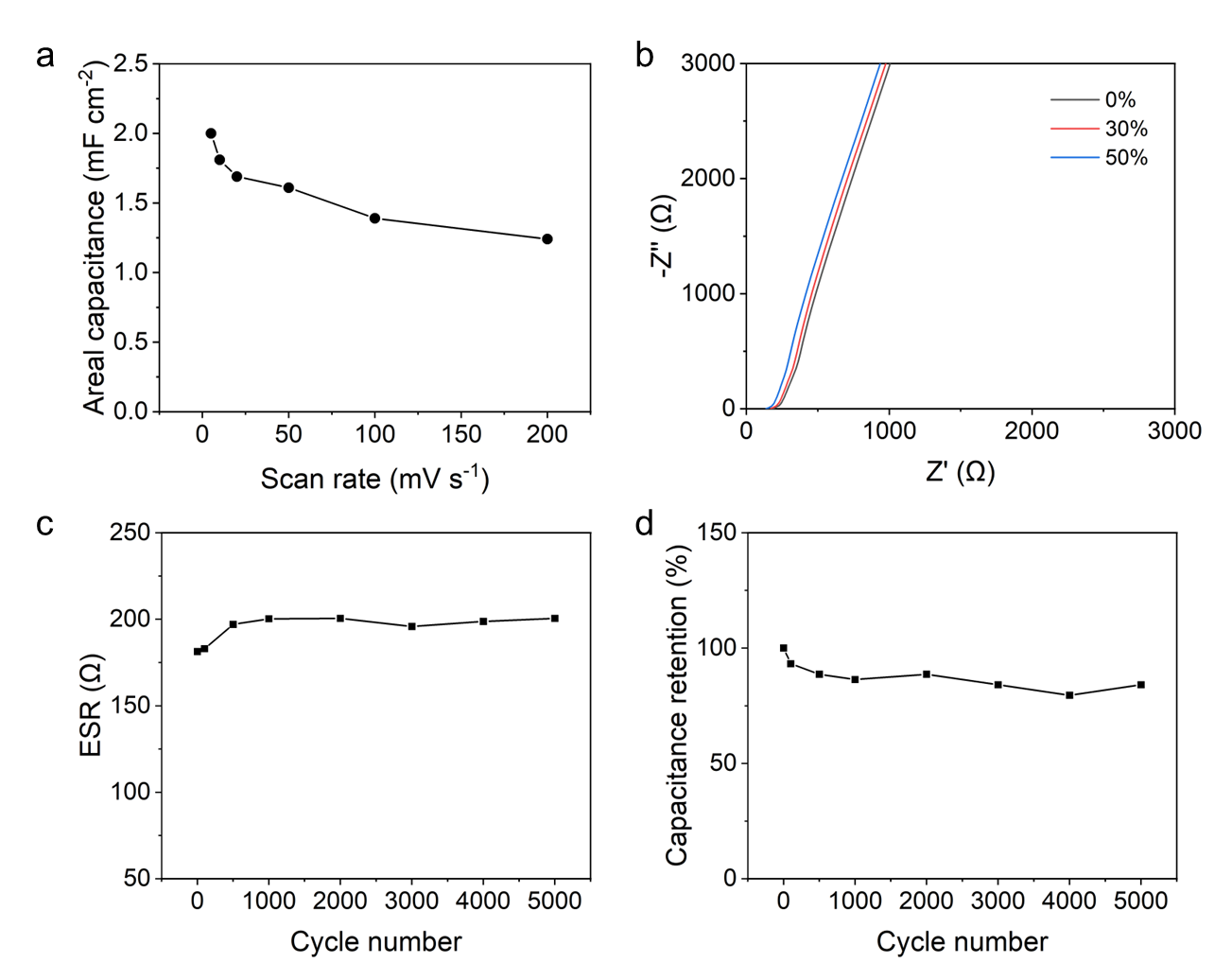


**Fig. S27** **a** Areal capacitance of the MSCs as a function of scan rate based on the CV curves. **b** Nyquist plots of the MSC (active area: 1 cm^2^) under tensile strains of 0%, 30%, and 50%. **c** Evolution of equivalent series resistance (ESR) measured after different numbers of charge−discharge cycles. **d** Cycling stability of the MSC over 5000 charge−discharge cycles at a current density of 0.5 mA cm^-2^, showing the capacitance retention


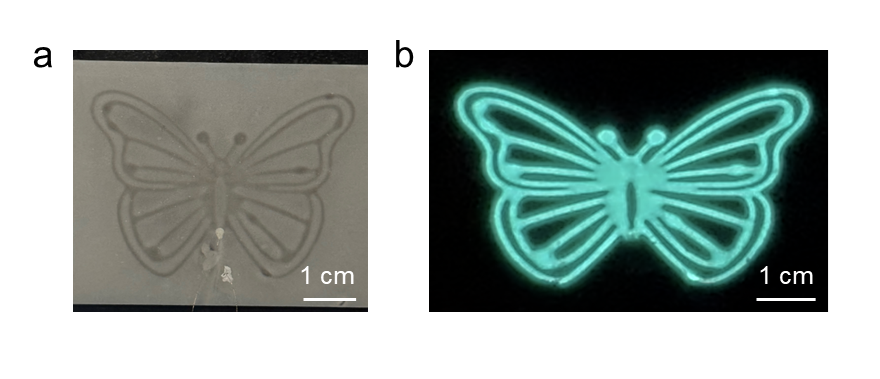


**Fig. S28** Photographs of (**a**) electroluminescent devices with butterfly patterns, and (**b**) the devices operating under 20% strain, driven by an AC field of 2.9 V/μm at 1 kHz


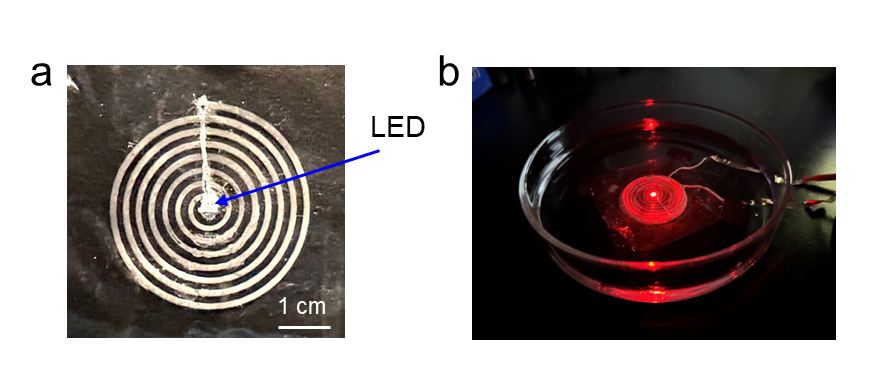


**Fig. S29** Photographs of (**a**) a wireless coil integrated with an LED, and (**b**) the coil wirelessly transmitting energy in a glass dish filled with water


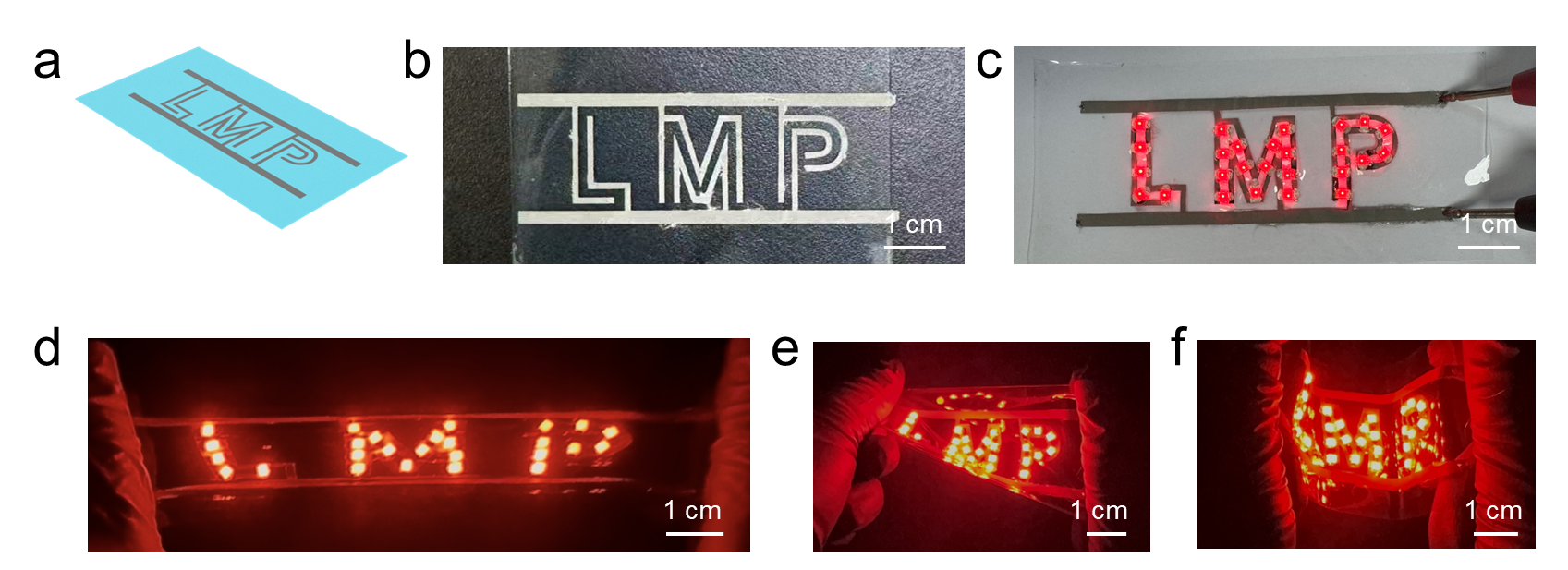


**Fig. S30** **a** Schematic illustration of the circuit layout of the stretchable display. **b** Optical image of the MLHMs circuit line stencil-printed on a TPU film. **c** Photograph of the display illuminated under a 3 V DC voltage. **d-f** Photographs of the stretchable display operating under (d) stretching, (e) twisting, and (f) bending

**Table S1** Summary of the performance of printable and stretchable conductors

| Materials | Conductivity (S/m) | Stretchability (%) | Functionality | Refs. |
| --- | --- | --- | --- | --- |
| EGaIn/Ag flakes/EVA | 833100 | 1000 | Electrically conductive | [S1] |
| EGaIn/AgNPs | 485000 | 118 | Electrically conductive | [S2] |
| EGaIn/PVP | 380000 | 1000 | Electrically conductive | [S3] |
| EGaIn/PVA-AD/CD | 1580000 | 800 | Electrically conductive | [S4] |
| EGaIn/PSS | 1500000 | 500 | Electrically conductive | [S5] |
| EGaIn/CB/silicone elastomer | 5.6 | 286 | Electrically conductive | [S6] |
| EGaIn/𝜶-lipoic acid/choline chloride | 1500000 | 150 | Electrically conductive | [S7] |
| EGaIn/𝜷-cyclodextrin/sodium dodecyl sulfate | 340000 | 710 | Electrically conductive | [S8] |
| EGaIn/Carbopol | 500000 | 300 | Electrically conductive | [S9] |
| Ag particles  /MWCNTs/PDMS | 1160200 | 150 | Electrically conductive | [S10] |
| Ag flakese/vegetable oil polyurethane | 1283300 | 350 | Electrically conductive | [S11] |
| AgNps/MWCNTs/Ag flaks/PVDF copolymer | 557100 | 140 | Electrically conductive | [S12] |
| Ag flakes/fluorine rubber | 73800 | 215 | Electrically conductive | [S13] |
| Ag flakes/fluorine rubber | 616800 | 400 | Electrically conductive | [S14] |
| EGaIn/MXene/HPU | 370000 | 700 | Electrically conductive and electrochemically active | This work |

**Table S2** Comparison of the activation-strain of stretchable conductors based on EGaIn

| Materials | Activation-strain (%) | Refs. |
| --- | --- | --- |
| EGaIn/PVP | ~10 | [S3] |
| EGaIn/PVA-AD/CD | ~60 | [S4] |
| EGaIn/Carbopol | ~4 | [S9] |
| EGaIn/PVP | ~50 | [S15] |
| EGaIn/TPU | ~55 | [S16] |
| EGaIn/MXene/HPU | 2.5 | This work |

* To facilitate consistent comparison with other strain-activated LMP composites, activation strain is defined as the strain at which the conductor resistance falls below 100 Ω.

**Table S3** Performance comparison of MXene-based MSCs

| Electrode material | Electrolyte | Test  condition | Cycles/ Retention (%) | Stretchability (%) | Refs. |
| --- | --- | --- | --- | --- | --- |
| MXene | PVA/H_2_SO_4_ | 20 mV s^-1^ | 1200/90 | N/A | [S17] |
| MXene | PVA/H_2_SO_4_ | 20 mV s^-1^ | 10000/90 | N/A | [S18] |
| MXene | PVA/H_2_SO_4_ | 32 µA cm^-2^ | 24000/95 | N/A | [S19] |
| MXene/rGO | PVA/H_2_SO_4_ | 2 mA cm^-2^ | 15000/91 | N/A | [S20] |
| MXene | PVA/H_2_SO_4_ | 1 A g^-1^ | 10000/90 | N/A | [S21] |
| MXene | PVA/H_2_SO_4_ | 200 µA cm^-2^ | 10000/94 | N/A | [S22] |
| MXene | PVA/H_2_SO_4_ | 10 mA cm^-2^ | 5000/83 | N/A | [S23] |
| MXene/Graphene | PVA/H_3_SO_4_ | 200 mV s^-1^ | 2500/82 | N/A | [S24] |
| MXene | PVDF-HFP/  EMIMBF_4_ | 1.0 mA cm^-2^ | 2500/without fading | N/A | [S25] |
| MXene/LM | PVDF-HFP/  [EMIM][TFSI] | 0.5 mA cm^-2^ | 5000/84 | 50 | This work |

N/A, not applicable

**Supplementary References**

1. J. Wang, G. Cai, S. Li, D. Gao, J. Xiong et al., Printable superelastic conductors with extreme stretchability and robust cycling endurance enabled by liquid-metal particles. Adv. Mater. **30**(16), e1706157 (2018). <https://doi.org/10.1002/adma.201706157>
2. M. Tavakoli, M.H. Malakooti, H. Paisana, Y. Ohm, D.G. Marques et al., EGaIn-assisted room-temperature sintering of silver nanoparticles for stretchable, inkjet-printed, thin-film electronics. Adv. Mater., e1801852 (2018). <https://doi.org/10.1002/adma.201801852>
3. L. Tang, L. Mou, W. Zhang, X. Jiang, Large-scale fabrication of highly elastic conductors on a broad range of surfaces. ACS Appl. Mater. Interfaces **11**(7), 7138–7147 (2019). <https://doi.org/10.1021/acsami.8b20460>
4. Q. Wang, X. Ji, X. Liu, Y. Liu, J. Liang, Viscoelastic metal-in-water emulsion gel *via* host–guest bridging for printed and strain-activated stretchable electrodes. ACS Nano **16**(8), 12677–12685 (2022). <https://doi.org/10.1021/acsnano.2c04299>
5. G.-H. Lee, Y.R. Lee, H. Kim, D.A. Kwon, H. Kim et al., Rapid *Meniscus*-guided printing of stable semi-solid-state liquid metal microgranular-particle for soft electronics. Nat. Commun. **13**, 2643 (2022). <https://doi.org/10.1038/s41467-022-30427-z>
6. M. Singh, P. Bhuyan, S. Jeong, S. Park, Directly printable, non-smearable and stretchable conductive ink enabled by liquid metal microparticles interstitially engineered in highly entangled elastomeric matrix. Adv. Funct. Mater. **35**(2), 2412178 (2025). <https://doi.org/10.1002/adfm.202412178>
7. K. Zhao, Y. Zhao, J. Wang, C. Ye, Liquid metal nanoparticle-based multifunctional printable electronics enabled by polymerizable deep eutectic solvents. Adv. Funct. Mater. **35**(24), 2424490 (2025). <https://doi.org/10.1002/adfm.202424490>
8. J. Qiu, R. Yu, X. Du, T. Zhou, Y. Chen et al., Liquid metal gel ink with self-activating conductivity for 3D printing of multifunctional electronics. Small **21**(29), e2502722 (2025). <https://doi.org/10.1002/smll.202502722>
9. Z. Lin, X. Qiu, Z. Cai, J. Li, Y. Zhao et al., High internal phase emulsions gel ink for direct-ink-writing 3D printing of liquid metal. Nat. Commun. **15**, 4806 (2024). <https://doi.org/10.1038/s41467-024-48906-w>
10. B. Lee, H. Cho, S. Moon, Y. Ko, Y.-S. Ryu et al., Omnidirectional printing of elastic conductors for three-dimensional stretchable electronics. Nat. Electron. **6**(4), 307–318 (2023). <https://doi.org/10.1038/s41928-023-00949-5>
11. J. Lv, G. Thangavel, Y. Xin, D. Gao, W.C. Poh et al., Printed sustainable elastomeric conductor for soft electronics. Nat. Commun. **14**(1), 7132 (2023). <https://doi.org/10.1038/s41467-023-42838-7>
12. Chun, KY, Oh, Y, Rho, J, Ahn, JH, Kim, YJ et al, Highly conductive, printable and stretchable composite films of carbon nanotubes and silver. Nat. Nanotechnol. **5**(12), 853–857 (2010). <https://doi.org/10.1038/nnano.2010.232>
13. N. Matsuhisa, M. Kaltenbrunner, T. Yokota, H. Jinno, K. Kuribara et al., Printable elastic conductors with a high conductivity for electronic textile applications. Nat. Commun. **6**, 7461 (2015). <https://doi.org/10.1038/ncomms8461>
14. N. Matsuhisa, D. Inoue, P. Zalar, H. Jin, Y. Matsuba et al., Printable elastic conductors by *in situ* formation of silver nanoparticles from silver flakes. Nat. Mater. **16**(8), 834–840 (2017). <https://doi.org/10.1038/nmat4904>
15. R.-M. Chin, Y. Han, M.H. Malakooti, Surface-engineered liquid metal particles for printing stretchable conductive composites with enhanced stability under different strain rates. Adv. Mater. Technol. **9**(14), 2301324 (2024). <https://doi.org/10.1002/admt.202301324>
16. J. Chen, X. Chen, Y. Su, B. Shen, W. Zheng, Multifunctional strain-activated liquid-metal composite films with electromechanical decoupling for stretchable electromagnetic shielding. Mater. Horiz. **11**(24), 6381–6390 (2024). <https://doi.org/10.1039/d4mh00774c>
17. J. Orangi, F. Hamade, V.A. Davis, M. Beidaghi, 3D printing of additive-free 2D Ti_3_C2T_x_ (MXene) ink for fabrication of micro-supercapacitors with ultra-high energy densities. ACS Nano **14**(1), 640–650 (2020). <https://doi.org/10.1021/acsnano.9b07325>
18. H. Tetik, J. Orangi, G. Yang, K. Zhao, S. Bin Mujib et al., 3D printed MXene aerogels with truly 3D macrostructure and highly engineered microstructure for enhanced electrical and electrochemical performance. Adv. Mater. **34**(2), e2104980 (2022). <https://doi.org/10.1002/adma.202104980>
19. C.J. Zhang, B. Anasori, A. Seral-Ascaso, S.-H. Park, N. McEvoy et al., Transparent, flexible, and conductive 2D titanium carbide (MXene) films with high volumetric capacitance. Adv. Mater. **29**(36), 1702678 (2017). <https://doi.org/10.1002/adma.201702678>
20. Y. Yue, N. Liu, Y. Ma, S. Wang, W. Liu et al., Highly self-healable 3D microsupercapacitor with MXene-graphene composite aerogel. ACS Nano **12**(5), 4224–4232 (2018). <https://doi.org/10.1021/acsnano.7b07528>
21. W. Yang, J. Yang, J.J. Byun, F.P. Moissinac, J. Xu et al., 3D printing of freestanding MXene architectures for current-collector-free supercapacitors. Adv. Mater. **31**(37), e1902725 (2019). <https://doi.org/10.1002/adma.201902725>
22. C.J. Zhang, M.P. Kremer, A. Seral-Ascaso, S.-H. Park, N. McEvoy et al., Stamping of flexible, coplanar micro-supercapacitors using MXene inks. Adv. Funct. Mater. **28**(9), 1705506 (2018). <https://doi.org/10.1002/adfm.201705506>
23. H. Huang, H. Su, H. Zhang, L. Xu, X. Chu et al., Extraordinary areal and volumetric performance of flexible solid-state micro-supercapacitors based on highly conductive freestanding Ti_3_C_2_T*_x_* films. Adv. Electron. Mater. **4**(8), 1800179 (2018). <https://doi.org/10.1002/aelm.201800179>
24. H. Li, Y. Hou, F. Wang, M.R. Lohe, X. Zhuang et al., Flexible all-solid-state supercapacitors with high volumetric capacitances boosted by solution processable MXene and electrochemically exfoliated graphene. Adv. Energy Mater. **7**(4), 1601847 (2017). <https://doi.org/10.1002/aenm.201601847>
25. S. Zheng, C. Zhang, F. Zhou, Y. Dong, X. Shi et al., Ionic liquid pre-intercalated MXene films for ionogel-based flexible micro-supercapacitors with high volumetric energy density. J. Mater. Chem. A **7**(16), 9478–9485 (2019). <https://doi.org/10.1039/C9TA02190F>
